# Identifying multi-hit carcinogenic gene combinations: Scaling up a weighted set cover algorithm using compressed binary matrix representation on a GPU

**DOI:** 10.1038/s41598-020-58785-y

**Published:** 2020-02-06

**Authors:** Qais Al Hajri, Sajal Dash, Wu-chun Feng, Harold R. Garner, Ramu Anandakrishnan

**Affiliations:** 1 Department of Electrical and Computer Engineering, Virginia Tech, Blacksburg, VA 24060 USA; 2 Department of Computer Science, Virginia Tech, Blacksburg, VA 24060 USA; 3 Department of Biomedical Sciences, Edward Via College of Osteopathic Medicine, Blacksburg, VA 24060 USA; 4 Gibbs Cancer Center and Research Institute, Spartanburg, SC 29303 USA

**Keywords:** Computational models, Computational science, Computer science

## Abstract

Despite decades of research, effective treatments for most cancers remain elusive. One reason is that different instances of cancer result from different combinations of multiple genetic mutations (hits). Therefore, treatments that may be effective in some cases are not effective in others. We previously developed an algorithm for identifying combinations of carcinogenic genes with mutations (multi-hit combinations), which could suggest a likely cause for individual instances of cancer. Most cancers are estimated to require three or more hits. However, the computational complexity of the algorithm scales exponentially with the number of hits, making it impractical for identifying combinations of more than two hits. To identify combinations of greater than two hits, we used a compressed binary matrix representation, and optimized the algorithm for parallel execution on an NVIDIA V100 graphics processing unit (GPU). With these enhancements, the optimized GPU implementation was on average an estimated 12,144 times faster than the original integer matrix based CPU implementation, for the 3-hit algorithm, allowing us to identify 3-hit combinations. The 3-hit combinations identified using a training set were able to differentiate between tumor and normal samples in a separate test set with 90% overall sensitivity and 93% overall specificity. We illustrate how the distribution of mutations in tumor and normal samples in the multi-hit gene combinations can suggest potential driver mutations for further investigation. With experimental validation, these combinations may provide insight into the etiology of cancer and a rational basis for targeted combination therapy.

## Introduction

Cancer is one of the leading causes of death in the US with a projected 606,880 deaths in 2019^1^. Despite significant progress, effective treatment in advanced cases remain elusive, with most progress coming from prevention and early detection^2,3^. One of many possible reasons is that, although cancer is known to be caused primarily by multiple genetic mutations^4–9^, we cannot in general determine the specific combination of mutations responsible for a given instance of cancer^10,11^. Knowing the specific combination of hits in individual cases would allow us to develop more effective targeted combination therapies^10,11^. Although there are other factor that may contribute to cancer growth, such as tumor microenvironment, epigenetic modifications, gene fusion, germline defects, etc., the focus of this work is on somatic mutations^12–18^.

Current computational approaches search for cancer genes and mutations that increase cancer risk (the probability of getting cancer)^18–29^. In addition to other considerations, these methods search for genes that are significantly more frequently mutated in tumor samples compared to an estimated background mutation rate. However, mutations in any one of these genes by themselves do not always result in cancer, suggesting that carcinogenesis may require additional mutations, as illustrated by the following exmples. Germline mutations in BRCA genes increase the risk of breast cancer. Although 72% of women with this mutation are likely to be diagnosed with breast cancer by age 80, the other 28% of women are unlikely to get the disease^4^. In addition, none of the women with this inherited mutation are likely to get cancer before age 20, suggesting that additional genetic defects (hits) may be required for carcinogenesis. Similarly, individuals with APC mutations have a 7% risk of developing familial adenomatous polyposis, representing colon cancer predisposition, by age 21, which increases to a 99% risk by age 80^5^. The Li Fraumeni syndrome is another example where germline P53 mutations are associated with early onset cancer predisposition (e.g. soft tissue and bone sarcomas). However, cancer penetrance is less than 20% for children while approaching 80% by age 70, indicating that multiple hits are required for carcinogenesis^6–8^. The classic example for the multi-hit hypothesis is the study of retinoblastoma by Knudson^9^. The study showed that mutations in a single copy of the RB1 gene increases the risk of retinoblastoma, but a second mutation in the other copy of the gene is required for carcinogenesis. Mathematical models based on cancer incidence and mutation data suggest that combinations of two–eight oncogenic mutations are required for carcinogenesis, depending on cancer type^30–37^. *In vitro* studies have investigated the role of combinations of multiple genetic defects^38–40^. However, these studies consider known combinations, and are not designed to discover novel combinations.

Unlike computational approaches that search for driver mutations and cancer genes that increase the risk of cancer^18–29^, we previously developed an approach that explicitly searches for combinations of genes with mutations that are likely to be the cause of specific instances of cancer^41^. A set of 2-hit combinations identified by the algorithm was able to differentiate between tumor and normal tissue samples with 90% overall sensitivity and 93% overall specificity. Here, we extend the algorithm to identify combinations of three or more hits, since most cancers are estimated to require more than two hits^30–37^. However, the computational complexity of the algorithm, which is $O({G}^{h}\times C\times ({N}_{t}+{N}_{n}))$, limits the combinations that can be practically identified to 2-hit ($h=2$) combinations, where $G\approx 20000$ is the number of genes with mutations in the input data, *C* is the number of combinations identified by the algorithm, ${N}_{t}$ is the number of input tumor samples, ${N}_{n}$ is the number of input normal samples, and *h* is the number of hits. For example, it took 39 minutes to calculate a set of 2-hit combination for breast cancer (BRCA) using 911 tumor samples from the cancer genome atlas (TCGA). The algorithm was run on an Intel Xeon E5-2630 2.1 GHz central processing unit (CPU) with 256 GB memory. We estimate (as described in Methods) that it will take 253 days to calculate a set of 3-hit ($h=3$) combinations for BRCA, without any additional optimization or parallelization.

The goal of this work is to optimize the multi-hit algorithm to identify combinations of more than two hits in a practical time frame (<1 month). Achieving this level of speedup requires parallel execution across a large number of processors. Graphical processing units (GPUs) with thousands of processors are a natural choice for massively parallel processing^42^. However, GPUs have three key limitations that must be addressed to achieve significant speedup. (1) Speed of memory access is significantly slower on GPUs compared to CPUs, e.g. on the Intel Xeon E5-2630 CPU L1 and L2 cache access require 4 and 11 cycles respectively^43^, compared to 28 and 193 cycles for the NVIDIA V100 GPU^44^. Therefore, speedup from parallelization will be offset by slower memory access for algorithms that require access to a large amount of data from memory. (2) GPUs have limited amount of accessible memory, e.g. 32GB for the NVIDIA V100, compared to 1.5TB for Intel Xeon E5-2630^45^. (3) On NVIDIA GPUs, divergent branching during execution will result in unbalanced processor load, which also limits the achievable speedup from parallelization^46–50^. To address these GPU limitations, we employed two general strategies. (1) We used a compressed binary representation for the Gene-Sample Mutation matrix (described in Methods), which reduced memory requirement by 16-fold and resulted in an average 10 fold speedup (see Results). (2) We restructured and optimized the algorithm for parallel execution on a NVIDIA Tesla V100 PCIe graphical processing unit by minimizing divergent branching in addition to other optimizations described in the Methods section. The compressed binary representation alone resulted in a 0.4–18 fold speedup for the 2-hit algorithm, compared to the original integer matrix, depending on cancer type. This additional speedup, and the associated increase in software complexity, was not necessary for the identification of 2-hit combinations, and insufficient by itself for the identification of 3-hit combinations on the CPU. However, the optimized GPU implementation combined with the compressed binary representation was 0.7–224 times faster than the original CPU based integer matrix implementation, for the 2-hit algorithm, depending on cancer type. The 3-hit algorithm was an estimated 29–33,690 times faster for the optimized GPU implementation compared to the original CPU implementation. For the breast cancer samples mentioned above, we were able to compute a set of 3-hit combinations in 23 minutes with the optimized GPU implementation compared to the estimated 253 days for the original CPU implementation. The set of 3-hit combinations identified using a randomly partitioned training set was able to differentiate between tumor and normal samples in separate test data with overall sensitivity of 90% (95% confidence interval (CI) = 88–91%) and overall specificity of 93% (95% CI = 92–94%). Despite this relatively high accuracy, the multi-hit gene combinations identified by our algorithm may not represent cancer genes (see Discussion). Further experimental validation will be required to determine if mutations within these genes may play a role in cancer genesis or progression.

The remainder of this manuscript is organized as follows. In the Results section, we describe the speedup achieved by the optimized parallel implementation, the breakdown of the contribution of different optimizations, and the accuracy of the multi-hit combinations identified. In the Discussion section, we illustrate how the distribution of somatic mutations in tumor and normal samples in the gene combinations can be used to identify potential driver mutations for further investigation. Our approach and results are summarized in the Conclusions. In the Methods section, we describe the multi-hit algorithm, the compressed binary representation of the input matrix, the mapping of the algorithm to the GPU, and its optimization for parallel execution.

## Results

Cancer is estimated to be caused by a combination of a small number of (two to eight) genetic mutations (hits)^30–37^. We had previously developed an algorithm for identifying a set of 2-hit combinations of genes with mutations, that was able to differentiate between tumor and normal samples with high sensitivity and specificity^41^. Due to its computational complexity the algorithm is impractical for identifying more than two hits^41^.

To identify combinations of more than two hits, we restructured and optimized the algorithm for parallel execution on a GPU, as described in the Methods section. These modifications can be grouped into two broad categories: compressed binary matrix representation and GPU parallelization.

The compressed binary matrix optimization and GPU parallelization resulted in an average speedup of 12,144x for the 3-hit algorithm, relative to the original integer matrix based CPU implementation. With this speedup, we were able to identify 3-hit combinations for the 32 cancer types for which data was available in TCGA. In addition, we were able to identify 4-hit combinations for 14 cancer types for which the run time was less than 15 days. The accuracy of the 3-hit combinations was found to be comparable to the 2-hit combinations, with overall sensitivity of 90% (95% CI = 88–91%) and average specificity of 93% (95% CI = 92–94%).

### Optimization and parallelization reduces run time for the 2-hit algorithm

**Figure 1:**
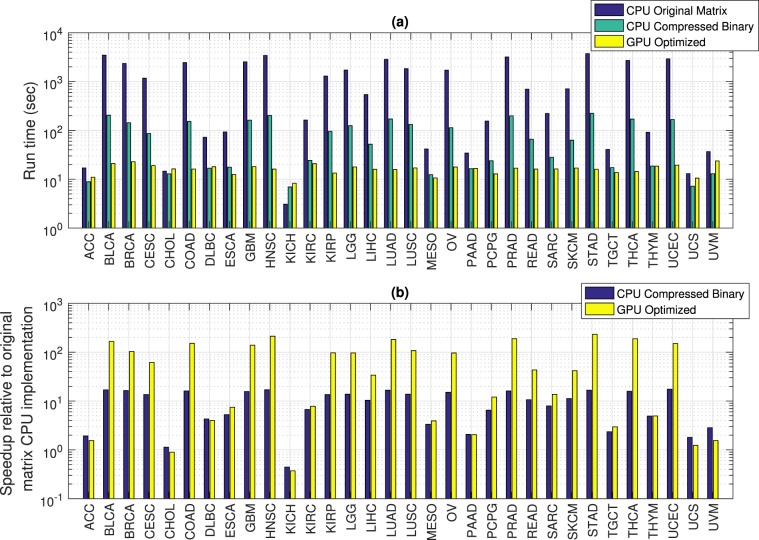
Comparison of different implementations of the multi-hit algorithm for identifying 2-hit combinations. (**a**) Run time for the original matrix implementation on the CPU ranges from 3–3723 sec compared to 7–223 sec for the compressed binary CPU implementation and 5–33 sec for the optimized GPU implementation. (**b**) Speedup is on average 10-fold for the compressed binary CPU implementation and 68-fold for the optimized GPU implementation compared to the original matrix CPU implementation. Names for the cancer types shown along the x-axis are listed in Table S1.

Figure 1(a) shows that the run time for identifying 2-hit combinations ranges from 5–33 sec for the optimized GPU implementation compared to 7–223 sec for the compressed binary CPU implementation and 3–3,723 sec for the original matrix CPU implementation. The optimized GPU implementation of the 2-hit algorithm is on average 68 times faster than the original CPU implementation, with the speedup ranging from 0.7–224x (Fig. 1(b)). However, due to the relatively large fixed data load time, these speedup numbers understate the effect of the optimization and parallelization described in the Methods. On average, the data load time for the 2-hit optimized GPU implementation is 85% of the total run time. The speedup values for the 3-hit algorithm, where the above average data load times are 14% of total run time for the optimized GPU implementation, is more closely representative of the effect of optimization and parallelization. Detailed run times for each cancer type, with a breakdown of the data load time, for different implementations of the 2-hit algorithm are shown in Supplementary Table S2.

### Run time reduction permits identification of 3-hit combination

**Figure 2:**
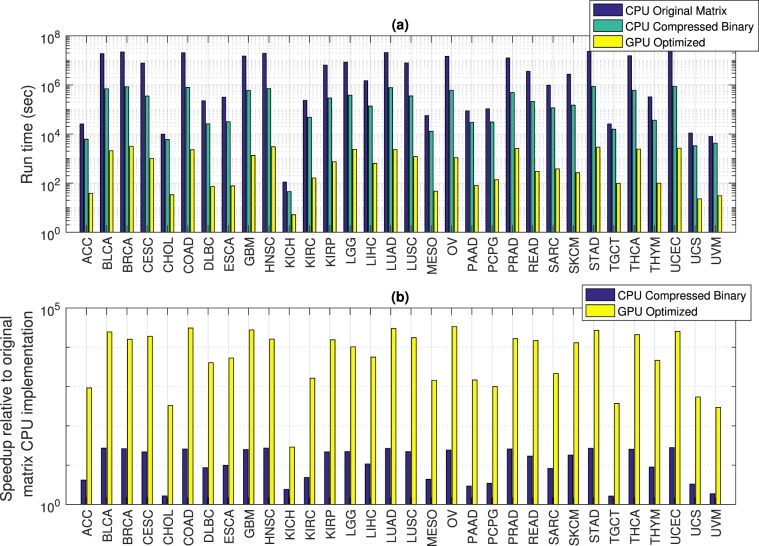
Comparison of different implementations of the multi-hit algorithm for identifying 3-hit combinations. (**a**) Run time for the original matrix CPU implementation ranges from 110 sec to an estimated 282 days, compared to 46 sec to 10 days for the compressed binary CPU implementation and 4 sec to 23 min for the optimized GPU implementation. Run times for the original matrix CPU implementation requiring over 30 days were estimated as described in Methods. (**b**) Speedup for the compressed binary CPU implementation ranged from 2x–28x, and from 29x–33,690x for the optimized GPU implementation. Names for the cancer types shown along the x-axis are listed in Table S1.

Figure 2(a) shows that the run time for identifying 3-hit combinations ranges from 4 sec to 23 min for the optimized GPU implementation compared to 46 sec to 10 days for the compressed binary CPU implementation. For the original integer matrix CPU implementation, the run time ranges from 110 sec to an estimated 282 days. The optimized 3-hit algorithm on the GPU results in an estimated 29 –33,690 fold speedup compared to the estimated time for the original matrix based CPU implementation (Fig. 2(b)), with an average 12,144 fold estimated speedup. Detailed run times and speedup for each cancer type for different implementations of the 3-hit algorithm are shown in Supplementary Information Tables S4 and S5.

### Run time reduction permits identification of some 4-hit combination

With the reduction in run time resulting from the optimization and parallelization described in the Methods section below, we were able to identify 4-hit combinations for some cancer types. For cancer types where the number of genes with mutations $G < 19000$, it takes less than 15 days to identify 4-hit combinations. Detailed run times for these cancer types are shown in Supplementary Information Table S6. To identify 4-hit combinations for all cancer types, additional optimization and parallelization across multiple GPUs will be required, which will be presented in a separate forthcoming study.

### Contribution of optimization techniques to overall speedup

**Figure 3:**
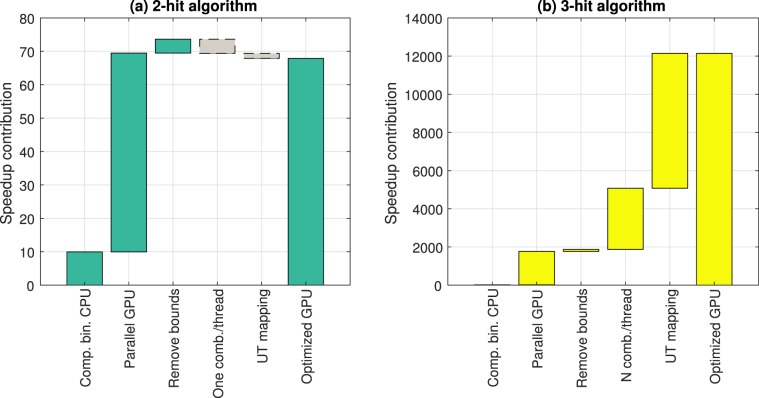
Average contribution of optimizations and parallelization to speedup. Breakdown of contributions due to compressed binary representation, GPU parallelization, removal of branch and bound logic, single two-gene combination per thread, and mapping of upper triangular (UT) gene combination to a sequential thread ID. (**a**) Breakdown of 2-hit speedup. (**b**) Breakdown of 3-hit combinations. Contribution due to compressed binary representation is 15x for 3-hits which is not visible in the scale of the figure.

The speedup reported above results from five key enhancements: compressed binary representation of the Gene-Sample Mutation matrices, parallel execution across multiple GPU cores, removal of branch and bound logic, computation of a single two-gene combination per thread, and mapping upper triangular matrix of two-gene combinations to thread index. See Methods section below. The breakdown of the contribution due to each of these enhancements is shown in Fig. 3. The speedup contribution of each enhancement is calculated as the difference in average speedup for the implementation of each enhancement compared to the original matrix CPU implementation See Supplementary Tables S3 and S5. On average, the largest contribution to speedup for the 2-hit algorithm is due to GPU parallelization (Fig. 3(a)). The largest contribution for the 3-hit algorithm is due to mapping GPU threads to the upper triangular matrix of two-gene combinations (Fig. 3(b)). The contribution due to the first three factors – compressed binary representation, GPU parallelization and removal of branch and bound logic – is roughly consistent between the 2-hit and 3-hit algorithms. However, the enhancements for a single two-gene combination per thread and upper triangular thread mapping slow down the 2-hit algorithm. This is because, for the 2-hit algorithm, speedup due to higher processor utilization from these enhancements are offset by the additional operations and global memory access required to implement these modifications.

### Multi-hit combinations differentiate between tumor and normal samples with high accuracy

**Figure 4:**
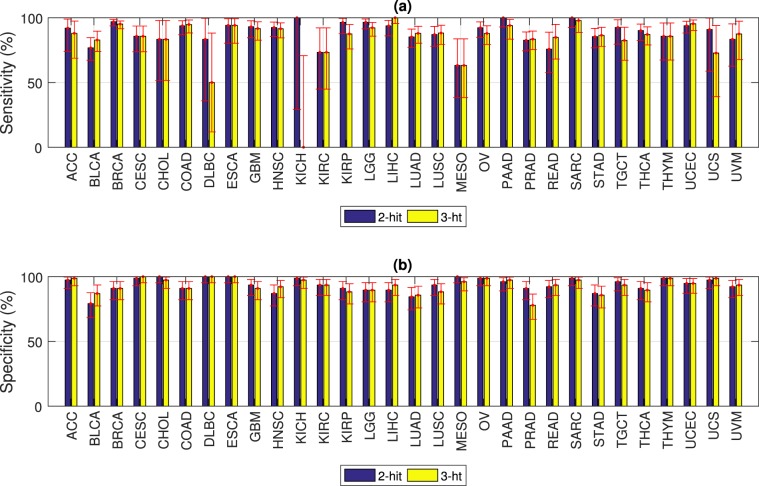
Accuracy of 2- and 3-hit combinations. (**a**) Sensitivity varies from 63–100% for 2-hit combinations, and from 50–100% for 3-hit combinations, excluding KICH for which there were only a total of 9 tumor samples. (**b**) Specificity varies from 79–100% for 2-hit combinations, and from 78–100% for 3-hit combinations. Sensitivity and specificity were calculated on a randomly selected 25% Test data set. Error bars represent 95% CI. Cancer types with relatively large 95% CI (CHOL, DLBC, KICH, KIRP, MESO and UCS) are due to small sample size (total of 44, 43, 9, 88, 69 and 46 samples respectively).

The 3-hit combinations identified using a 75% randomly selected Training set identified an average of 7 combinations per cancer type with a total of 335 unique genes, compared to 8 combinations per cancer type with a total of 310 unique genes for the 2-hit combinations. The identified combinations are listed in Supplementary Tables S7–S9. The 3-hit combinations were able to differentiate between tumor and normal samples in a separate Test set with overall sensitivity of 90% (95% CI = 88–91%) and overall specificity of 93% (95% CI = 92–94%), as shown in Fig. 4(b). This was comparable to the overall sensitivity and specificity for 2-hit combinations with sensitivity = 90% (95% CI = 89–92%) and specificity = 94% (95% CI = 93–95%), as shown in Fig. 4(a). The difference in average sensitivity and specificity between 2- and 3-hit combinations was −6% (95% CI = −13.5–+1.5%) and −1% (95% CI = −3.6–+1.6%) respectively, with corresponding p-values of 0.12 and 0.44 respectively. Accuracy values are listed in Supplementary Tables S10 and S11. Since we did not see any improvement in accuracy for 3-hit combinations compared to 2-hit combinations, we speculate that additional accuracy improvement will require examining individual mutations within genes, as discussed below.

## Discussion

Not all mutations within a cancer gene are oncogenic^22,23,25,28^. However, to make the problem of identifying multi-hit combinations tractable, the algorithm searched through all possible gene combinations, instead of all possible combinations of mutations. In the tumor sample data used, there were over 400,000 unique somatic mutations across ~20,000 genes. It is theoretically possible to search all possible combinations of 400,000 protein altering somatic mutations instead of combinations of 20,000 genes with somatic mutations. However, searching all possible combinations of 400,000 mutations would increase the computational complexity for identifying 3-hit combinations by over six orders of magnitude, making the problem computationally intractable. In addition, since there can be multiple different carcinogenic mutations within a gene, combinations of individual mutations will occur less frequently than combinations of genes with mutations, further increasing the challenge of identifying carcinogenic combinations within this much larger set of possible combinations. Therefore, we chose to first focus on combinations of genes with mutations. Mutations within these gene combinations can then be examined to identify potential driver mutations for further investigation, as illustrated below.

**Figure 5:**
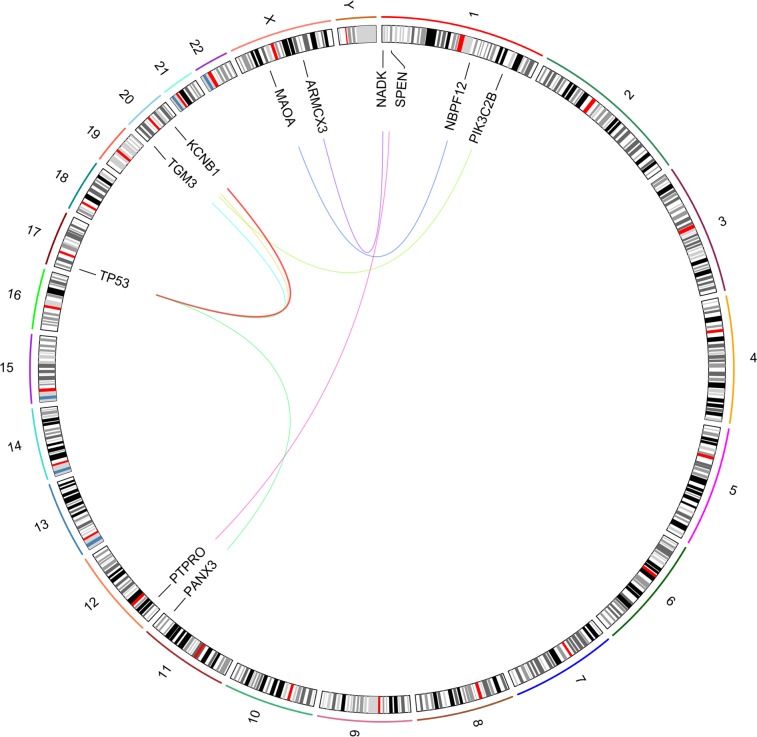
2-hit combinations identified for ovarian serous cystadenocarcinoma (OV). The outer circle shows individual chromosomes with corresponding ideograms shown in the inner circle. Genes that comprise 2-hit combinations are labeled inside the circle. Each 2-hit combination is identified by differently colored lines connecting two genes. The red line represents the gene combination discussed in further detail. This image was  generated using RCircos^87^.

**Figure 6:**
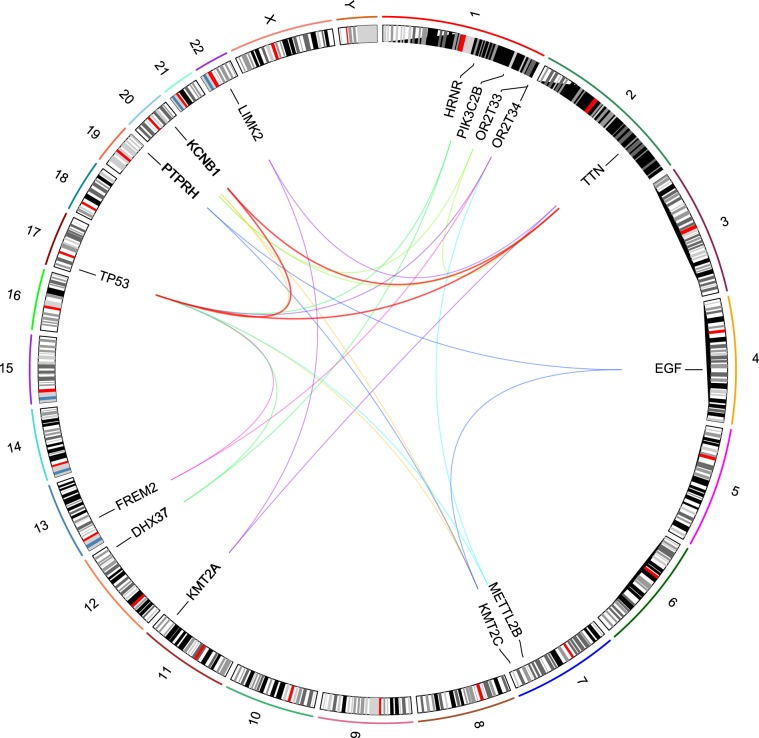
3-hit combinations identified for ovarian serous cystadenocarcinoma (OV). The outer circle shows individual chromosomes with corresponding ideograms shown in the inner circle. Genes that comprise 3-hit combinations are labeled inside the circle. Each 3-hit combination is identified by differently colored lines connecting three genes. The red line represents the gene combination discussed in further detail. This image was generated using RCircos^87^.

**Figure 7:**
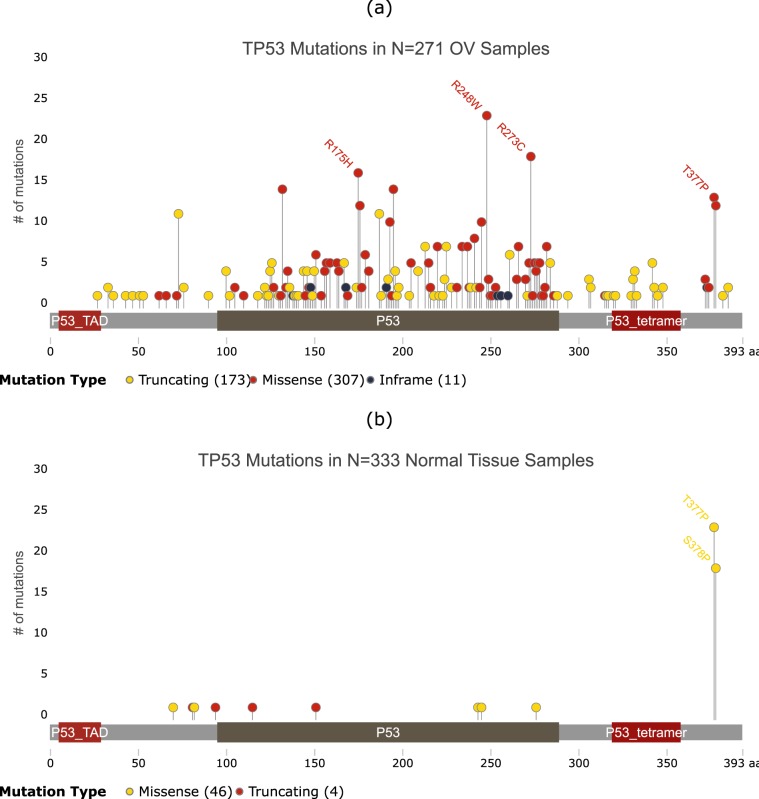
Distribution of somatic mutations in TP53 in ovarian tumor samples and normal samples. The horizontal bar shows amino acid position within the protein, with labels showing known functional domains. Vertical lines show the number of samples with protein altering mutations at each amino acid position. The most frequently mutated sites for each gene in (**a**) tumor and (**b**) normal samples are labeled for comparison. Image generated using g3viz^88^.

**Figure 8:**
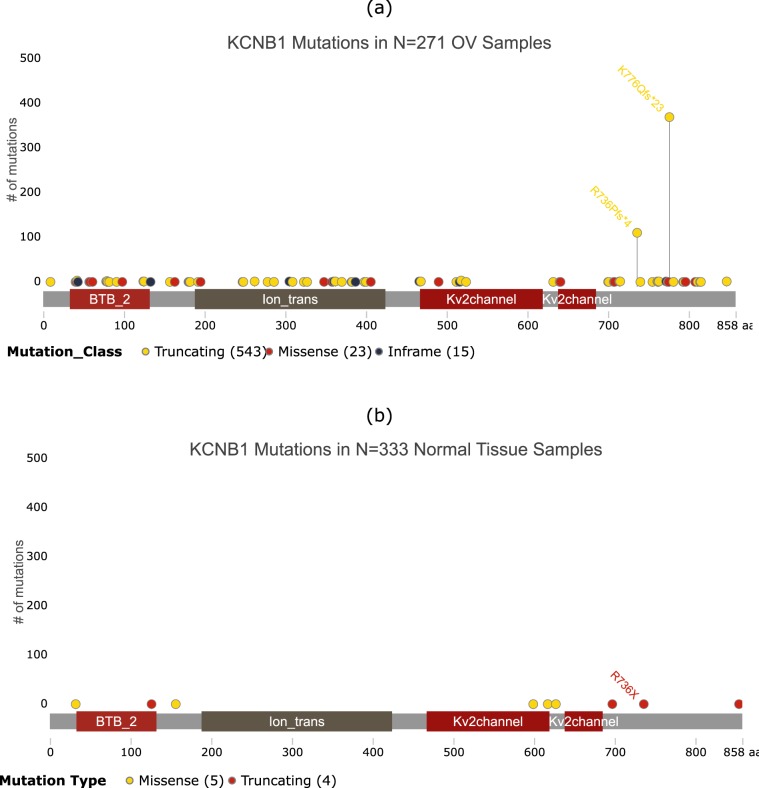
Distribution of somatic mutations in KCNB1 in ovarian tumor samples and normal samples. The horizontal bar shows amino acid position within the protein, with labels showing known functional domains. Vertical lines show the number of samples with protein altering mutations at each amino acid position. The most frequently mutated sites for each gene in (**a**) tumor and (**b**) normal samples are labeled for comparison. This image was generated using g3viz^88^.

**Figure 9:**
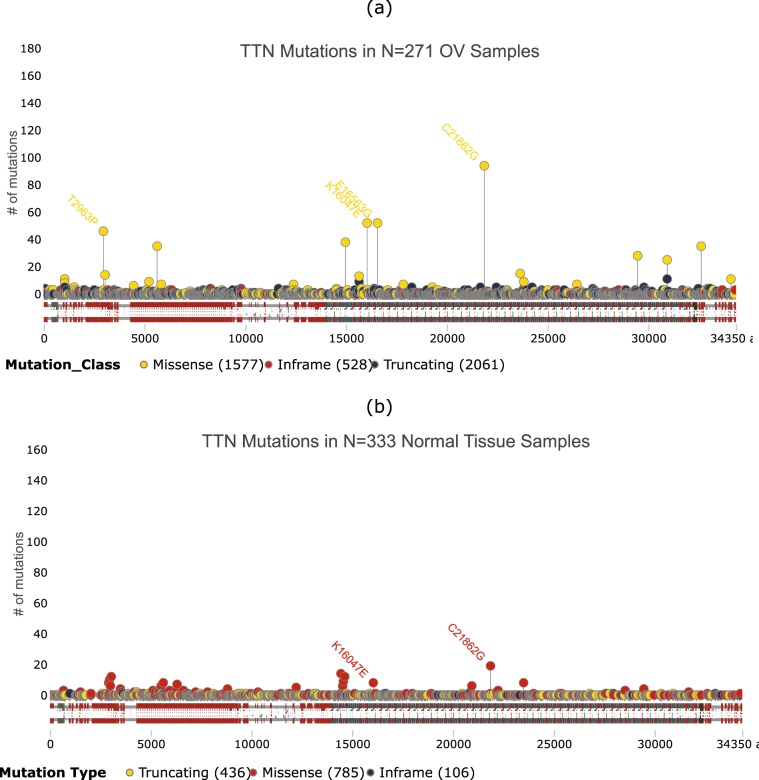
Distribution of somatic mutations in TTN in ovarian tumor samples and normal samples. The horizontal bar shows amino acid position within the protein, with labels showing known functional domains. Vertical lines show the number of samples with protein altering mutations at each amino acid position. The most frequently mutated sites for each gene in (**a**) tumor and (**b**) normal samples are labeled for comparison. This image was generated using g3viz^88^.

Consider for example, the 2- and 3-hit combinations identified for ovarian serous cystadenocarcinoma (OV) (Figs. 5 and 6). The most commonly occurring 2- and 3-hit combination are TP53+KCNB1 and TP53+KCNB1+TTN respectively. Mutations in TP53 and KCNB1 occur in 279 of 317 OV tumor samples and mutations in TP53, KCNB1 and TTN occur in 271 of 317 OV tumor samples. The distribution of protein altering somatic mutations in TP53, KCNB1 and TTN for the 271 OV tumor samples containing mutations in all three genes are shown in Figs. 7(a), 8(a) and 9(a), respectively. The distribution of protein altering somatic mutations in TP53, KCNB1 and TTN for 333 normal samples are shown in Figs. 7(b), 8(b) and 9(b), respectively. The difference in the frequency of individual mutations between tumor and normal samples may suggest potential driver mutations for further investigation.

The TP53 gene codes for the Tumor Protein P53. Mutations in TP53, a tumor suppressor gene, have been extensively implicated in many cancers, including OV^51–56^. In the 271 OV tumor samples containing the TP53+KCNB1+TTN 3-hit combination, TP53 contains on average 1.8 protein altering somatic mutations per sample, compared to 0.15 mutations per sample in normal samples, with clear differences in the distribution of these mutations (Fig. 7). The three most frequently occurring mutations in the tumor samples (amino acid positions R248, R273, and R175) are potential driver mutations, since they rarely occur in normal tissue (Fig. 7). The mutation frequency at R248, R273 and R175 are 0.08, 0.07 and 0.06 per tumor sample, compared to 0.00 per normal sample (p-value < 0.0001 for the difference in proportions). In fact, previous studies have shown that the R248W, R273H and R175H mutations not only cause a loss of P53-based tumor suppressor activity, but also result in genomic instability causing gain of oncogenic activity^57–59^. On the other hand the two most frequently mutated amino acid positions in normal samples, T377 and T378, are likely to be passenger mutations. Normal tissue mutation frequencies of 0.07 and 0.05 per normal sample are comparable to tumor tissue mutation frequencies of 0.04 and 0.05 per tumor sample for T377 and T378, respectively (Fig. 7).

The KBNB1 gene codes for the Potassium Voltage-Gated Channel Subfamily B Member 1 protein. KCNB1 has been previously identified as a prognostic factor in gliomas due to its tumor suppressor function^60^. It contains on average 2.14 protein altering somatic mutations per tumor sample in the 271 OV samples containing the TP53+KCNB1+TTN 3-hit combination, compared to 0.03 mutations per normal sample (p-value < 0.0001) (Fig. 8). The two most frequently occurring mutations at K776 and R736 are potential driver mutations worthy of further investigation. The mutation frequencies at these positions are 1.37 and 0.41 per tumor sample compared to 0.00 and 0.003 per normal sample, respectively (Fig. 8). Although KCNB1 has been extensively studied, primarily in the context of epilepsy^61–66^, these studies do not include either of the two mutations identified here. These two mutations occur in the unstructured C-terminus cytoplasmic tail region of this transmembrane potassium channel protein^61,66^. Further *in vitro* investigation will be required to understand how these mutations may affect the expression, structure or function of this protein, to determine if these could be driver mutations.

The TTN gene codes for the Titin protein of striated muscle. TTN expression level has been previously identified as prognositc marker for Ewing’s sarcoma^67^, and TTN mutations have been associated with several myopathies^68–72^. Titin is a large protein consisting of 34,350 amino acids, with a correspondingly large number of mutations, 15.37 protein altering somatic mutations per tumor sample and 3.98 mutations per normal sample, on average (Fig. 9). Three of the most frequent mutations in TTN in tumor samples, C21862G, E1656G and T2963P, occur more frequently in tumor samples compared to normal samples, suggesting that these may be potential driver mutations that should be investigated further. The mutation frequencies at these amino acid positions are 0.17, 0.20 and 0.20 per tumor sample, compared to 0.06, 0.003, and 0.03 per normal tissue sample, respectively (Fig. 9). Although TTN mutations have been extensively studied, primarily in the context of myopathies^69–72^, these studies do not include any of the three mutations identified here.

The above only provide a starting point for further investigation. The positive selection implied by the higher mutation frequencies seen above are confounded by several factors, including tumor microenvironment, tissue and cell type, epigenetic modifications, gene expression and co-expression, etc.^12–18^. A more detailed analysis of the potential driver mutations identified above using available literature, gene expression data, copy number variation, associated pathways, functional annotation, protein localization, etc., could provide additional evidence to either support or reject the mutation as a driver mutation. This information can be iteratively incorporated into the search algorithm described in Methods. We expect that excluding likely passenger mutations will reduce the number of false positives and prioritizing likely driver mutations will reduce false negatives, improving the accuracy of the combinations identified. However, this could also potentially limit the discovery of novel genes.

Note that these somatic mutations were calculated using *protected* whole exome sequencing data from tumor samples with matched blood-derived normal samples. For tumor samples, protected somatic mutation data (MAF files) were downloaded from the cancer genome atlas (TCGA) with permission. Somatic mutations for normal tissue samples with matched blood-derived normal samples were called using the same protocol used by TCGA, as described in the methods. Variants called using matched blood-derived normal data identifies significantly more mutations than the number of variants called without matched blood-derived normal samples, for the following reasons^73,74^. Biopsy specimens contain a mix of tumor and normal tissue cells, tumor-infiltrating lymphocytes, and stromal cells. In addition, tumor cells themselves can be genetically diverse. As a result, mutations in a subset of the cell population will present at a relatively low frequency. Using blood derived normal samples as a reference allows for the identification of such low frequency variants. Variants that could potentially lead to de-identification of donors (~80 million variants) are considered “protected” data in TCGA, and are not accessible by tools such as cBioPortal and TCGA queries that are based on “open” access data (~3 million variants). For example, the protected TCGA MAF files contain 617 protein-altering somatic mutations in TP53 in 317 OV samples, compared to the 276 somatic mutations reported by cBioPortal using open access data^75^.

## Conclusion

Cancer is caused by a combination of a small number of genetic defects (hits), estimated to be in the range of two to eight. However, the specific multi-hit combination for each instance of cancer can be different, even for the same type of cancer. Existing approaches focused on identifying individual cancer genes can not identify the specific multi-hit combination responsible for an individual instance of cancer. We previously developed a fundamentally different approach focused on identifying multi-hit combinations. Due to the O(*G*^*h*^) scaling of the search algorithm, where $G\approx 20000$ is the number genes and *h* is the number of hits, we were limited to identifying 2-hit combinations. In this work we present optimization and parallelization techniques that allowed us to extend the algorithm to identify 3-hit combinations, and some 4-hit combinations. The 3-hit combinations are able to differentiate between tumor and normal samples with overall 90% sensitivity (95% CI = 88–91%) and 93% specificity (95% CI = 92–94%). We illustrate how the distribution of somatic mutations in these genes can be used to identify potential driver mutations for further investigation. For example, we identified two protein-altering somatic mutations in the KCNB1 gene which occur significantly more frequently in TCGA ovarian cancer samples compared to normal samples (p-value < 0.0001), suggesting that these mutations may be positively selected for in ovarian cancer. However, further experimental validation is required to determine if these mutations represent novel cancer driver mutations, or are simply passenger mutations. The muti-hit combinations identified here, with experimental validation, can be used to identify the specific cause of individual instances of cancer, allowing for the rational design of more effective targeted combination therapies.

## Methods

The multi-hit algorithm identifies combinations of genes with mutations that may represent the potential cause for individual instances of cancer. Due to its computational complexity, the algorithm was limited to identifying combinations of two hits. To identify combinations of more than two hits, the algorithm was restructured and optimized for parallel execution as described below.

### The multi-hit algorithm

**Figure 10:**
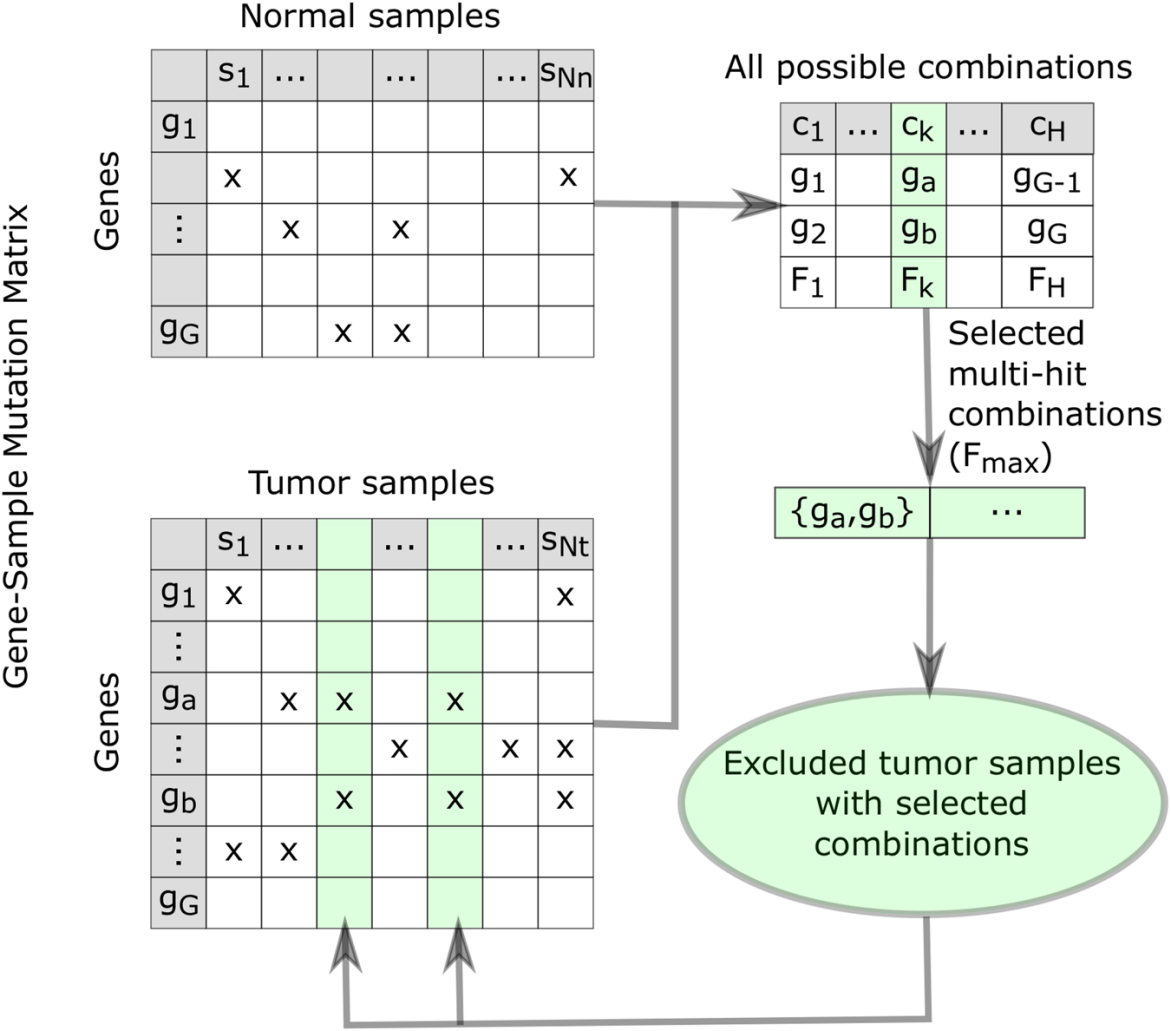
Algorithm for finding multi-hit combinations, illustrated for 2-hit combinations. The cells marked with x in the Gene-Sample Mutation matrices represent samples with mutations in the corresponding gene. There are $H=G(G-1)/2$ possible 2-hit combinations involving two different genes. The algorithm iterates through three steps. (1) Eq. (1) is used to calculate *F*_*i*_ for each combination. (2) The combination (*g*_*a*_ and *g*_*b*_ in this example) with the maximum value of *F*_*i*_, (*F*_*k*_ in this example) is added to the list of selected multi-hit combinations. (3) Tumor samples containing mutation in the selected combination of genes are excluded from consideration in subsequent iterations of the algorithm. The green shaded columns in the Tumor Gene-Sample Mutation matrix represent excluded samples in the iteration shown. The algorithm terminates when all tumor samples have been excluded, i.e. “covered” by the set of multi-hit combinations.

The problem of identifying a set of multi-hit combinations of genes with mutations that are most likely to be responsible for individual instances of cancer can be mapped to a weighted set cover (WSC) problem^41^. The WSC problem falls into a class of “NP-complete” problems for which there is no known polynomial-time solution, but given a solution it can be verified in polynomial time^76,77^. In addition, problems in this class can be mapped to each other in polynomial time, such that if a polynomial time solution is found for any one of these problems, all of these problems can be solved in polynomial time^78,79^. Although the WSC problem is computationally intractable for our problem size, a near-optimal approximate solution can be found using a greedy algorithm. A greedy algorithm for the WSC problem was adapted for the problem of identifying a set of multi-hit combinations as previously described^41^. To summarize, the algorithm iterates through the following three steps until all tumor samples have been excluded, as illustrated in Fig. 10.

1. Compute a weighted accuracy metric ${F}_{i}$ for all $i=[1,H]$ possible *h*-hit combinations, where *H* is the number of possible combinations. ${F}_{i}$ is a combined measure of the specificity and sensitivity with which each combination can differentiate between tumor and normal samples in a training set.
  1 $${F}_{i}=\frac{\alpha T{P}_{i}+T{N}_{i}}{{N}_{t}+{N}_{n}}$$
  where, for a given combination *i*, $T{P}_{i}$ is the number of true positives (tumor samples with mutations in the gene combination *i*), $T{N}_{i}$ is the number of true negatives (normal samples without mutations in the gene combination *i*), ${N}_{t}$ is the total number of tumor samples, ${N}_{n}$ is the total number of normal samples and $\alpha =0.1$ is a weighting factor to balance the contribution of sensitivity and specificity to the metric.
2. Select the combination of hits with the maximum *F*_*i*_ value, and add it to the list of selected multi-hit combinations.
3. Exclude all tumor samples that contain mutations in this combination of genes, from further consideration.

The computational complexity of the algorithm is $O({G}^{h}\times C\times ({N}_{t}+{N}_{n}))$ where *G* is the number of genes and *C* is the number of combinations selected. The input to the algorithm are two Gene-Sample Mutation matrices, a tumor mutation matrix $({M}_{ij}^{t})\in {\{0,1\}}^{G\times {N}_{t}}$ and a normal mutation matrix $({M}_{ij}^{n})\in {\{0,1\}}^{G\times {N}_{n}}$. Non zero values in these binary matrices represent mutations in gene *g*_*i*_, $i=[1,G]$ within sample ${s}_{j}$, $j=[1,{N}_{t}]$ for tumor samples and $j=[1,{N}_{n}]$ for normal samples (Fig. 10). In addition, these are sparse matrices with only 2% of the elements having a non-zero values. To take advantage of these characteristics of the input matrices, we considered two possible alternatives to the matrix representation: indexed array and compressed binary representations, as described below.

### Indexed array data structure

One option for speeding up the above algorithm’s runtime, is to replace the Gene-Sample Mutation matrices with an indexed array data structure. The indexed array data structure reduces the number of arithmetic operations by excluding samples in the gene that do not have mutations. The data structure consists of two arrays. One is a samples array where samples with mutations within each gene are listed sequentially. The second is a gene index array, which contains the starting index into the samples array for each gene. With the indexed array representation, the algorithm would only examine samples with mutations in the genes being considered, instead of all samples. It is more efficient than the original matrix representation since samples that do not contain mutations in a gene are not evaluated. On average only 2% of samples have mutations for a given gene, therefore, we expected a significant speedup with an indexed list representation However, due to an increase in the number of instructions and divergent branches, the speedup using this data structure was less than what was achieved using the compressed binary representation described below.

### Compressed binary representation

**Figure 11:**
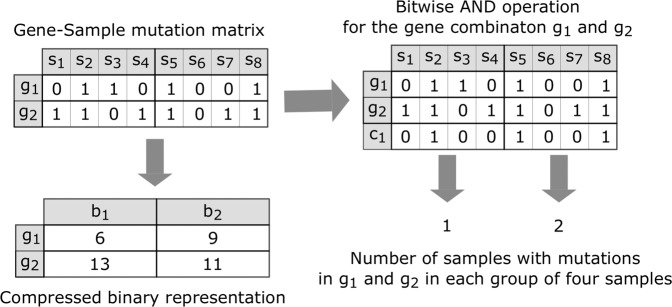
Compressed binary representation and bitwise operation for determining the number of samples with mutations in a combination of two genes. *Left:* Compressed binary representation of Gene-Sample Mutation matrices, illustrated for a 4-bit unsigned integer. *s*_*i*_ represents the normal or tumor samples shown in Fig. 10. Elements with 0 in the matrix indicate that the sample does not contain mutations in the corresponding gene, while 1 indicates that the sample does contain a mutation in the corresponding gene. Mutations in four samples can be represented by a single 4-bit unsigned integer. *Right:* Given any two genes *g*_*i*_, *g*_*j*_, the number of samples containing mutations in both these genes is determined by a bitwise AND operation for each of the integers representing mutations in *g*_*i*_ with the corresponding set of integers for *g*_*j*_, and then counting the number of non-zero bits.

The binary values in the Gene-Sample Mutation matrices permits a reduction in memory requirement using a compressed binary representation. In addition, bitwise operations can be used with the compressed binary representation to reduce computational cost and divergent branching (discussed in section 5.5.2). Figure 11 illustrates how mutations in a group of four samples can be compressed into four bits. In the original implementation, each Gene-Sample value was represented as a single 16-bit short integer^41^. For this implementation, we represent groups of 64 samples as a single 64-bit unsigned integer, which requires 4-fold fewer vector operations compared to the 16-bit unsigned integer representation. The resulting speedup was confirmed experimentally (results not shown). The compressed binary representation also results in 16-fold reduction in memory since each word of memory stores data for 16 samples, compared to one sample per word in the original integer matrix representation.

The number of samples with mutations in a combination of genes can then be efficiently determined by a bitwise *AND* operation followed by a count of the non-zero bits, as illustrated in Fig. 11. To count the number of non-zero bits for the CPU code, we implemented Brian Kernighan’s algorithm^80^. For the GPU implementation, we used the built-in popcll() function to count the number of bits set to 1 in the 64 bit unsigned integer. This function was faster than our own implementation of Brian Kernighan’s algorithm (e.g. run time for optimized GPU implementation for the 3-hit algorithm for BRCA using popcll(), is 451 sec faster than the run time using Kernighan’s algorithm).

For the computation of 2-hit combinations for breast cancer (BRCA), the compressed binary representation resulted in a 16-fold speedup on the CPU compared to the original matrix representation, as shown in the Results. Since this speedup was considerably larger than the corresponding 4-fold speedup for the indexed array representation described above, we did not consider the indexed array structure any further.

### Mapping to the NVIDIA Tesla V100 PCIe graphical processing unit (GPU)

**Figure 12:**
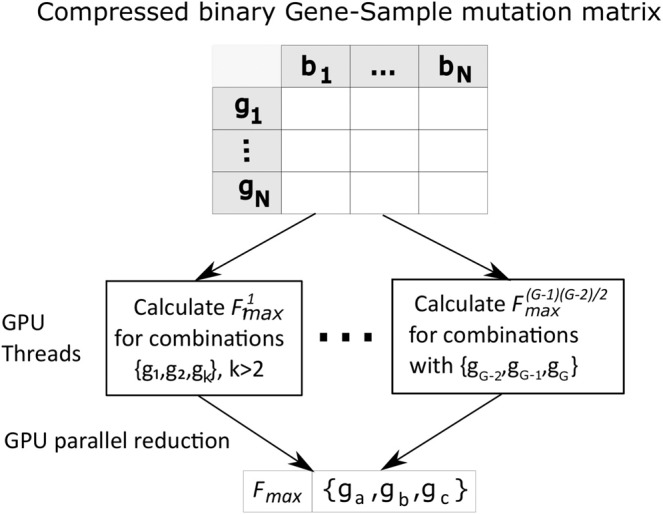
Mapping the multi-hit CPU algorithm to the GPU, illustrated for the 3-hit algorithm with the compressed binary representation (Fig. 11). Each GPU thread computes ${F}_{max}^{i}$ for a subset of all possible combinations. The results of each thread is stored in GPU global memory. *F*_*max*_ across all subsets of combinations is calculated using parallel reduction^81^.

To further speed up the multi-hit algorithm, we restructured the CPU code for parallel execution on one GPU, specifically the NVIDIA Tesla V100 PCIe GPU^42^. The V100 consists of 5376 32-bit floating point cores, 5376 32-bit integer cores, 2688 64-bit floating point cores, 672 tensor cores and 336 texture units. The cores are partitioned into 84 streaming multiprocessors (SM) with 128 KB of shared memory per SM, 6 MB of *L2* cache and 32 GB of global memory for the GPU. Each SM is further partitioned into four single instruction multiple thread (SIMT) *warps*, i.e. the same instruction is executed on all cores within a warp, with each core running a different thread^42^. For parallel execution of the algorithm, we partition the computation of *F*_*max*_ across multiple threads, where each thread computes the maximum value of *F* for a subset of combinations (${F}_{{\max }}^{i}$) where $i\in [1,G(G-1)/2]$. For 2-hit combinations, each thread processes a single combination, therefore ${F}_{max}^{i}={F}^{i}$ for the combination *i*, say ${g}_{a}$ and ${g}_{b}$ where $a < b\le G$. For 3-hit combinations ${F}_{{\max }}^{i}$ is the maximum value for all 3-hit combinations with two of the hits corresponding to 2-hit combination $i$, i.e. ${g}_{a}$, ${g}_{b}$ and ${g}_{c}$ where $a < b < c < G$, as illustrated in Fig. 12. The maximum value *F*_*max*_ across all ${F}_{{\max }}^{i}$ is then calculated using parallel reduction^81^.

The sequential implementation of the above algorithm for 3-hit combinations is illustrated in Algorithm 1. The for loops in lines 7, 8, and 9, in the sequential algorithm iterates through all possible $$(\begin{array}{c}G\\ 3\end{array})$$ 3-hit combinations. Lines 10–14 compute *F* for one such combination. Lines 16–18 compute overall best combination along with it’s *F*_*max*_ value. The remaining part of the algorithm updates excluded samples.

To run on parallel compute units of a GPU, we modified the above sequential algorithm as illustrated in Algorithm 2. We combined the two outer for loops into a single one, which iterates $$(\begin{array}{c}G\\ 2\end{array})$$ times (Line 7). Each iteration of this combined for loop can run in parallel on a different GPU compute units. For each of these parallel tasks, indexed by *λ*, there is a sequential for loop (Line 11) which computes the best combination among the 3-hit combinations that start with *i*, *j* corresponding to the *λ* (2). The mapping of *λ* to *i* and *j* in lines 7 and 8 is described below under “Minimizing divergent branches”. At the end of this outer for loop, our parallel algorithm performs a parallel reduction (Line 25) to compute the best combination^81^. Then the tumor samples covered by this best combination are added to the covered samples.

This parallelization allows us to run $$(\begin{array}{c}G\\ 2\end{array})$$ parallel tasks with load $O(G({N}_{t}+{N}_{n}))$ instead of $$(\begin{array}{c}G\\ 3\end{array})$$ sequential tasks with load $O({N}_{t}+{N}_{n})$.

**Algorithm 1:**
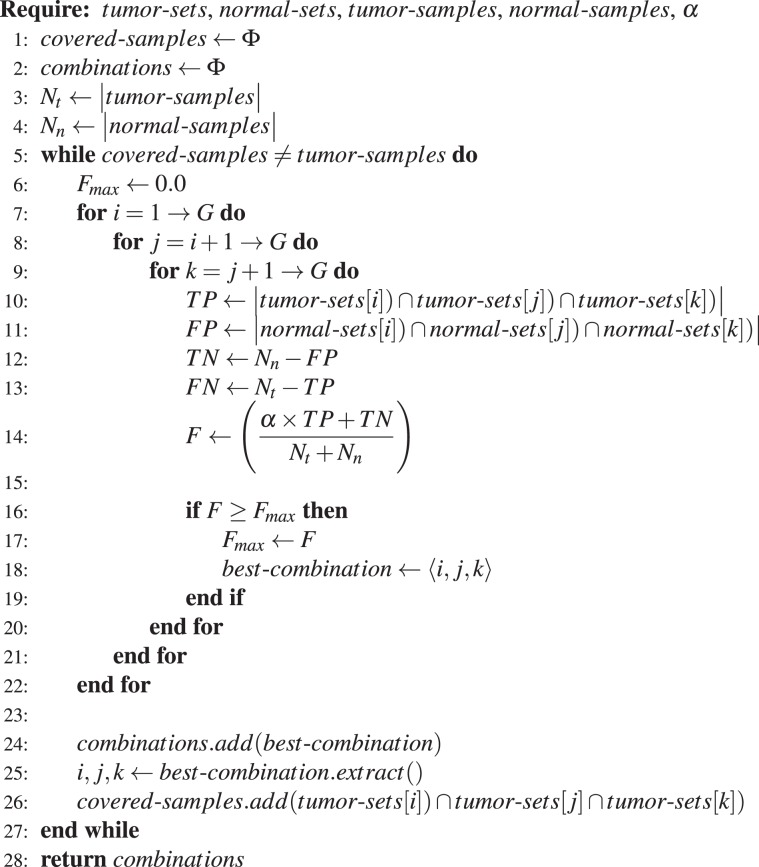
Sequential algorithm to compute 3-hit combinations.

### GPU optimization

The differences between the CPU and GPU architectures make certain coding techniques, that may be appropriate for serial execution on a CPU, sub-optimal or even incorrect for parallel execution on a GPU. Three critical considerations for minimizing processor latency are: synchronizing update access to memory locations shared by multiple processors, divergent branching in a single instruction multiple thread (SIMT) architecture, and relative speed of shared memory vs. global memory.

#### Minimizing GPU synchronization

A straightforward implementation would have a single global memory location for *F*_*max*_ which could be updated by all thread. However, such an implementation would require a synchronization or locking protocol to maintain cache coherence^82^. Synchronization of GPU threads to ensure correct results introduced significant processor latency and resulted in the GPU implementation running slower than the CPU version. We therefore allocate a separate memory location for each thread *i* to store its ${F}_{{\max }}^{i}$ value (Fig. 12), avoiding the need for synchronized memory access. We then use parallel reduction to efficiently calculate the global *F*_*max*_ value^81^.

**Algorithm 2:**
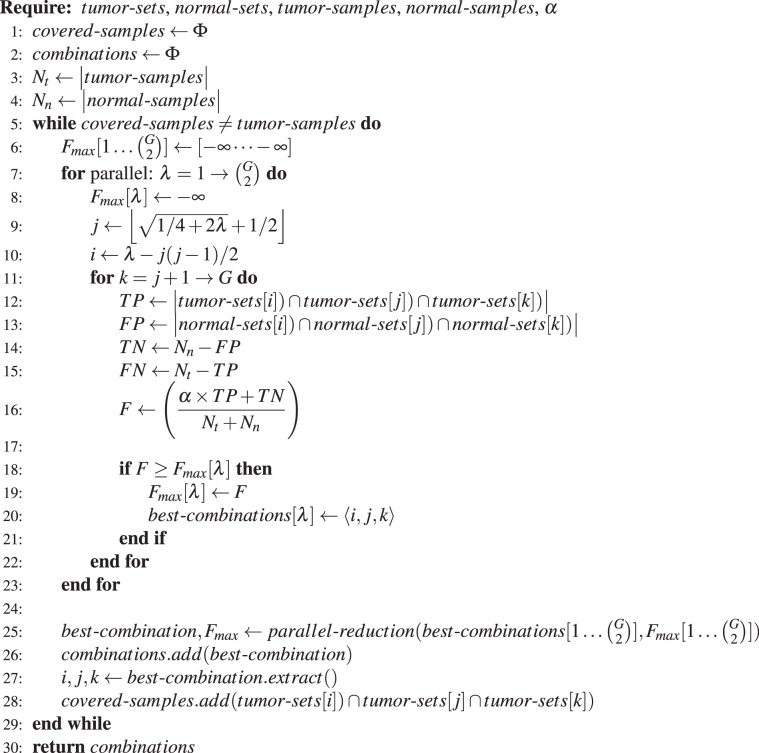
Parallel algorithm to compute 3-hit combinations.

#### Minimizing divergent branches

In the SIMT warps used by the GPU within streaming multiprocessors (SM), divergent branches introduce significant processor latency^46–50^. Divergent branches are IF-ELSE and LOOP control statements that cause execution along different paths depending on conditional values. Within a warp, all possible execution paths are serialized and evaluated^42,49,50^. Thus, significant latency is introduced due to the execution of instruction within branches that are not used. In the original integer matrix CPU implementation, conditional statements are required to count the number of samples with mutations in a gene combination. In the compressed binary implementation, these conditional statements are replaced by a set of bitwise operations, as described above (Fig. 11). In addition, the CPU implementation calculates a bound on the maximum possible value for ${F}^{i}$ for a given combination $i$. If this value is less than the intermediate value for *F*_*max*_ (Eq. (1)), subsequent processing for the combination is skipped. Although this strategy improved performance on the CPU, eliminating this branch and bound logic on the GPU resulted in an additional 6% average speedup for the 2-hit algorithm and 3-hit algorithm (Supplementary Tables S3 and S5).

The multi-hit algorithm only considers combinations represented by the upper triangular matrix, i.e. combinations of ${g}_{i}$ and ${g}_{j}$ where $i < j$. In the CPU implementation, processing is limited to the upper triangular matrix by loop control conditions. To eliminate these conditional branches, using the formulation from ref. ^83^, modified for upper triangular matrix instead of lower triangular matrix, we map the thread index $\lambda$ to the upper triangular matrix $i < j$ as follows:

2 $$\begin{array}{rcl}j & = & \lfloor \sqrt{1/4+2\lambda }+1/2\rfloor \\ i & = & \lambda -j(j-1)/2\end{array}$$

#### Using shared memory for parallel reduction

The ${F}_{max}^{i}$ values calculated by each GPU thread *i* is stored in global memory (Fig. 12). The maximum value *F*_*max*_ across all these threads can be calculated using parallel reduction, directly in global memory. However, accessing global memory is significantly slower than accessing shared memory^44^. Therefore, we divide global memory data into blocks which are copied into shared memory. Parallel reduction is performed within each block using shared memory to compute the ${F}_{max}^{j}$ for block $j$. The result is copied back to a new allocation in global memory. This new allocation is 1024 (the number of virtual threads per block) times smaller than the original allocation. This process is repeated with the newly allocated values until the single *F*_*max*_ values has been calculated. The above approach reduced the total global memory used by approximately 50%, e.g. 2.87 GB for BRCA compared to 5.75 GB without this approach.

### Mutation data

Input data for the algorithm consists of two Gene-Sample Mutation matrices, one for tumor samples and another for normal samples. Each element ${M}_{ij}$ of the matrix is either 1 or 0 depending on if gene *i* has a protein altering (missense, nonsense, insertion or deletion) somatic mutation in sample *j*, or not. This information was calculated from whole exome sequencing data available from The Cancer Genome Atlas (TCGA) database^84^. Somatic mutation data, calculated using Mutect2^85^, for matched tumor and blood derived normal samples was available for download from TCGA in Mutation Annotation Format (MAF). For 333 normal samples with matched blood derived normal samples, we calculated somatic mutations using the same Mutect2 protocol. See ref. ^41^ for additional details.

### Speedup calculation

Speedup was calculated as ${t}_{ref}/{t}_{new}$ where ${t}_{new}$ and ${t}_{ref}$ are the run times for the new code and the baseline reference, respectively. Run time was determined using the Linux time command, with run time = sys time + user time. For identifying 2-hit combinations, the run time for the original CPU implementation^41^ was used as the baseline reference ${t}_{ref}$. However, this was not practical for 3-hit combinations for cancer types where the run time using the original matrix code was over 30 days. Therefore we estimated the run time for cancer types taking over 30 days, based on the actual run times for cancer types requiring less than 30 days. We assumed that the average ratio of 2-hit vs. 3-hit speedup for the compressed binary CPU implementation compared to the original matrix CPU implementation is the same for both categories (run time <30 days and run time >30 days). For cancer types with run time <30 days, we calculated the average ratio $R={\rm{Avg}}({S}_{3}/{S}_{2})$, where *S*_3_ is the 3-hit speedup and *S*_2_ is the 2-hit speedup, for the CPU compressed binary implementation compared to the CPU original matrix implementation. For cancer types with 3-hit run time >30 days, we estimated the run time as $R\cdot {S}_{2}\cdot {t}_{3cb}$, where ${t}_{3cb}$ is the 3-hit run time for the compressed binary CPU implementation. See Supplementary Table S4 for a list of actual and estimated 3-hit run times.

### Accuracy calculation

All available mutation data was randomly partitioned into two subsets, with 75% of the data (Training set) used to identify the multi-hit combination using the above algorithm. The remaining data (Test set) was used to calculate the sensitivity, specificity, and 95% confidence interval for the identified set of combinations’ ability to differentiate between tumor and normal samples. Sensitivity was calculated as *TP*/*N*_*t*_, where *TP* is the number of true positives (number of tumor samples containing one of the identified combinations) and *N*_*t*_ is the number of tumor samples. Specificity was calculated as *TN*/*N*_*n*_, where *TN* is the number of true negatives (number of normal samples without any of the identified combinations), and *N*_*n*_ is the number of normal samples. 95% confidence interval was calculated using the “exact” Clopper-Pearson method^86^. Overall sensitivity and specificity are calculated from the total count of true positives, true negatives, tumor samples, and normal samples for all cancer types, using the randomly selected 25% test data set. However, it is important to keep in mind that these multi-hit gene combinations may not represent cancer genes. Additional experimental validation is required to determine if mutations within these genes may play a role in cancer.

## Supplementary information

Supplementary Information.

## Data Availability

All source code for the multi-hit algorithm are available for download from https://bitbucket.org/qaisalhajri/multihit-gpu-implementation/src/master/.
