# Supplementary material for: Identifying multi-hit carcinogenic gene combinations: Scaling up a weighted set cover algorithm using compressed binary matrix representation on a GPU
Source: Sci Rep. 2020 Feb 6;10:2022. doi: 10.1038/s41598-020-58785-y (PMC7005272; doi:10.1038/s41598-020-58785-y)
Supplement: Supplementary file 1 — Supplementary Information. [file 41598_2020_58785_MOESM1_ESM.pdf]

Identifying multi-hit carcinogenic gene combinations: Scaling up a weighted set cover algorithm using compressed binary matrix representation on a GPU

Qais Al Hajri, Sajal Dash, Wu-chun Feng, Harold R Garner and Ramu Anandakrishnan

Supplementary Information

Table S1. Cancer type abbreviations with full names

Cancer Type	Cancer Name	Number of genes with mutations	Sample Size
ACC	Adrenocortical Carcinoma	9385	80
BLCA	Bladder Urothelial Carcinoma	18511	368
BRCA	Breast Invasive Carcinoma	19411	911
CESC	Cervical Squamous Cell Carcinoma and Endocervical Adenocarcinoma	18813	274
CHOL	Cholangiocarcinoma	11741	44
COAD	Colon Adenacarcinoma	19487	385
DLBC	Lymphoid Neoplasm Diffuse Large B-cell Lymphoma	14273	43
ESCA	Esophageal Carcinoma	15321	111
GBM	Glioblastoma Multiforme	18920	331
HNSC	Head and Neck Squamous Cell Carcinoma	18707	470
KICH	Kidney Chromophobe	2581	9
KIRC	Kidney Renal Clear Cell Carcinoma	15390	88
KIRP	Kidney Renal Papillary Cell Carcinoma	18117	228
LGG	Brain Lower Grade Glioma	17770	479
LIHC	Liver Hepatocellular Carcinoma	17592	312
LUAD	Lung Adenocarcinoma	18783	409
LUSC	Lung Squamous Cell Carcinoma	18454	305
MESO	Mesothelioma	10332	69
OV	Ovarian Serous Cystadenocarcinoma	18814	317
PAAD	Pancreatic Adenocarcinoma	15068	149
PCPG	Pheochromocytoma and Paraganglioma	11553	156
PRAD	Prostate Adenocarcinoma	17274	421
READ	Rectum Adenocarcinoma	17699	142
SARC	Sarcoma	17687	219
SKCM	Skin Cutaneous Melanoma	18236	92
STAD	Stomach Adenocarcinoma	18638	388
TGCT	Testicular Germ Cell Tumors	12989	149
THCA	Thyroid Carcinoma	18264	421
THYM	Thymoma	15291	107
UCEC	Uterine Corpus Endometrial Carcinoma	19889	495
UCS	Uterine Carcinosarcoma	8038	46
UVM	Uveal Melanoma	8231	73

Table S2. Run times for each cancer type and optimization/parallelization implementation for 2-hit combinations

	CPU Runtime (sec)		GPU Runtime (sec)				Data load time from run with "Upper Triangular Mapping"	% of Runtime
Cancer Type	Original Matrix	Compressed Binary	With Bound Check	Without Bound Check	2-Gene Comb per Thread	Upper Triangular Mapping		
ACC	17.04	8.86	10.23	10.97	10.67	11.92	9.48	79.55
BLCA	3465.21	204.81	13.49	20.90	19.82	15.50	12.78	82.45
BRCA	2349.10	143.22	22.54	22.80	20.99	21.12	19.57	92.65
CESC	1170.67	86.31	14.24	18.98	16.63	33.30	31.70	95.20
CHOL	14.63	12.89	10.44	16.28	13.20	15.60	14.17	90.83
COAD	2445.52	151.99	16.08	16.15	17.50	26.47	23.55	88.96
DLBC	72.37	16.84	11.17	18.06	12.90	12.13	10.01	82.48
ESCA	92.90	17.67	13.40	12.53	13.04	14.57	12.12	83.21
GBM	2534.09	161.98	25.14	18.21	17.33	21.59	18.73	86.74
HNSC	3426.28	201.69	18.68	16.12	25.28	15.83	12.96	81.88
KICH	3.08	6.93	6.41	8.28	5.95	4.59	3.20	69.72
KIRC	162.67	24.35	11.24	20.81	15.61	14.57	12.30	84.45
KIRP	1296.66	95.56	13.80	13.38	15.28	14.44	11.95	82.78
LGG	1721.05	124.80	21.44	17.86	17.54	17.08	14.74	86.28
LIHC	540.54	52.20	16.56	15.96	13.81	15.66	14.02	89.52
LUAD	2851.10	171.11	19.76	15.75	15.93	17.54	14.66	83.59
LUSC	1831.96	132.43	19.55	17.00	16.00	13.86	11.16	80.51
MESO	41.60	12.43	10.80	10.62	14.68	10.07	7.75	77.00
OV	1712.27	113.40	16.03	17.79	20.00	20.21	17.39	86.04
PAAD	34.26	16.47	14.34	16.70	12.86	16.89	14.71	87.12
PCPG	155.23	23.90	11.69	12.85	11.56	14.09	12.42	88.15
PRAD	3186.74	198.49	16.78	16.85	19.84	17.05	13.91	81.59
READ	699.30	65.92	15.99	16.17	18.89	18.41	15.89	86.29
SARC	222.34	28.09	25.30	16.25	16.40	17.79	16.32	91.75
SKCM	708.69	63.00	22.85	16.92	12.76	13.44	11.27	83.86
STAD	3722.63	222.74	20.14	16.00	15.94	24.82	22.76	91.68
TGCT	40.60	17.27	11.82	13.72	11.27	16.19	13.73	84.79
THCA	2702.23	170.36	13.70	14.33	19.82	21.20	19.42	91.59

THYM	91.54	18.61	22.54	18.51	18.48	17.45	15.22	87.21
UCEC	2916.98	166.65	40.02	19.40	20.10	19.52	17.73	90.83
UCS	13.02	7.22	13.78	10.54	15.45	9.95	7.81	78.51
UVM	36.73	12.94	9.02	23.67	9.47	16.17	13.89	85.87

Table S3: Speedup for each cancer type and opt/parallel implementation for 2-hit comb

	CPU Speedup	GPU Speedup			
Cancer Type	Compressed Binary	With Bound Check	Without Bound Check	2-Gene Comb per Thread	Upper Triangular Mapping
ACC	1.92	1.67	1.55	1.60	1.43
BLCA	16.92	256.87	165.80	174.83	223.56
BRCA	16.40	104.22	103.03	111.92	111.23
CESC	13.56	82.21	61.68	70.40	35.16
CHOL	1.13	1.40	0.90	1.11	0.94
COAD	16.09	152.08	151.43	139.74	92.39
DLBC	4.30	6.48	4.01	5.61	5.97
ESCA	5.26	6.93	7.41	7.12	6.38
GBM	15.64	100.80	139.16	146.23	117.37
HNSC	16.99	183.42	212.55	135.53	216.44
KICH	0.44	0.48	0.37	0.52	0.67
KIRC	6.68	14.47	7.82	10.42	11.16
KIRP	13.57	93.96	96.91	84.86	89.80
LGG	13.79	80.27	96.36	98.12	100.76
LIHC	10.36	32.64	33.87	39.14	34.52
LUAD	16.66	144.29	181.02	178.98	162.55
LUSC	13.83	93.71	107.76	114.50	132.18
MESO	3.35	3.85	3.92	2.83	4.13
OV	15.10	106.82	96.25	85.61	84.72
PAAD	2.08	2.39	2.05	2.66	2.03
PCPG	6.49	13.28	12.08	13.43	11.02
PRAD	16.05	189.91	189.12	160.62	186.91
READ	10.61	43.73	43.25	37.02	37.98
SARC	7.92	8.79	13.68	13.56	12.50
SKCM	11.25	31.01	41.88	55.54	52.73
STAD	16.71	184.84	232.66	233.54	149.99
TGCT	2.35	3.43	2.96	3.60	2.51
THCA	15.86	197.24	188.57	136.34	127.46
THYM	4.92	4.06	4.95	4.95	5.25
UCEC	17.50	72.89	150.36	145.12	149.44
UCS	1.80	0.94	1.24	0.84	1.31
UVM	2.84	4.07	1.55	3.88	2.27

Average	9.95	69.47	73.63	69.38	67.90
Difference		59.52	4.16	-4.25	-1.48

Table S4: Run times for each cancer type and optimization/parallelization implementation for 3-hit combinations

	CPU			GPU				Estimating 3-hit Runtime		
Cancer Type	Original Matrix (Actual)	Original Matrix (Estimated)	Compressed Binary	With Bound Check	Without Bound Check	2-Gene Comb per Thread	Upper Triangular Mapping	2-hit speedup	Actual 3-hit speedup	Speedup Ratio
ACC	25637.70		6154.37	242.95	230.90	38.40	27.77	1.92	4.17	2.17
BLCA	Est	18717738.63	690685.63	5288.89	5040.71	2084.68	762.80	16.92		
BRCA	Est	21898351.56	833525.77	7963.95	8113.89	3188.64	1371.28	16.40		
CESC	Est	7642749.79	351788.56	2742.86	2476.79	1004.30	400.08	13.56		
CHOL	9872.63		5995.75	211.42	191.85	33.74	29.99	1.13	1.65	1.45
COAD	Est	20290131.49	787288.85	4436.92	4000.58	2286.55	656.68	16.09		
DLBC	225396.82		26092.07	368.12	383.79	72.60	55.64	4.30	8.64	2.01
ESCA	316304.19		31764.47	403.31	373.36	77.06	59.31	5.26	9.96	1.89
GBM	Est	14897495.84	594508.51	3495.48	3342.66	1353.60	541.00	15.64		
HNSC	Est	19227116.11	706612.16	7732.98	8390.36	3027.58	1188.54	16.99		
KICH	110.07		45.51	9.70	11.05	5.19	3.82	0.44	2.42	5.44
KIRC	233983.25		48184.84	865.60	855.92	161.82	143.11	6.68	4.86	0.73
KIRP	Est	6333196.22	291392.53	2639.63	2667.77	740.82	408.90	13.57		
LGG	Est	8424102.26	381373.20	5368.93	6539.49	2385.62	820.28	13.79		
LIHC	1478483.54		137787.14	1663.09	1709.50	639.22	262.66	10.36	10.73	1.04
LUAD	Est	20618742.80	772557.06	4848.15	4334.81	2337.83	686.21	16.66		
LUSC	Est	7845996.18	354098.37	3248.13	3066.39	1205.87	445.01	13.83		
MESO	56329.84		12893.34	348.33	324.45	47.15	39.15	3.35	4.37	1.31
OV	Est	14579200.48	602809.32	2999.62	2725.97	1090.88	432.74	15.10		
PAAD	87607.16		29740.69	349.74	401.19	80.10	59.44	2.08	2.95	1.42
PCPG	106471.89		30772.91	998.31	952.82	138.62	106.26	6.49	3.46	0.53
PRAD	Est	12528692.60	487195.42	5932.85	5443.90	2555.16	758.79	16.05		
READ	Est	3568809.08	210030.53	1785.55	2296.18	303.79	241.22	10.61		
SARC	965960.28		116625.01	1333.41	1381.56	379.36	448.62	7.92	8.28	1.05
SKCM	Est	2699142.10	149801.25	1550.07	1397.64	264.81	207.69	11.25		
STAD	Est	23169148.34	865493.59	5813.69	5108.93	2933.15	861.64	16.71		
TGCT	25679.10		15717.66	563.57	571.38	97.61	68.89	2.35	1.63	0.69

THCA	Est	15294868.83	602000.04	4945.62	4558.58	2431.3	732.19
THYM	323881.19		36335.26	491.71	480.63	99.86	69.71
UCEC	Est	24363489.34	868994.04	8265.06	6343.07	2638.68	962.18
UCS	10969.19		3316.06	150.68	153.26	22.98	20.18
UVM	7948.31		4253.55	448.01	208.27	30.61	26.89

15.86		
4.92	8.91	1.81
17.50		
1.80	3.31	1.83
2.84	1.87	0.66

Average= 1.60

Table S5: Speedup for each cancer type and opt/parallel implementation for 3-hit combinations.

	CPU Runtime	CPU Speedup	GPU Speedup			
Cancer Type	Original Matrix	Compressed Binary	With Bound Check	Without Bound Check	2-Gene Comb per Thread	Upper Triangular Mapping
ACC	25637.7	4.17	105.53	111.03	667.65	923.22
BLCA	18717738.63	27.10	3539.07	3713.31	8978.71	24538.20
BRCA	21898351.56	26.27	2749.68	2698.87	6867.61	15969.28
CESC	7642749.786	21.73	2786.42	3085.75	7610.03	19103.05
CHOL	9872.63	1.65	46.70	51.46	292.61	329.20
COAD	20290131.49	25.77	4573.02	5071.80	8873.69	30898.05
DLBC	225396.82	8.64	612.29	587.29	3104.64	4050.99
ESCA	316304.19	9.96	784.27	847.18	4104.65	5333.07
GBM	14897495.84	25.06	4261.93	4456.78	11005.83	27536.96
HNSC	19227116.11	27.21	2486.38	2291.57	6350.66	16177.09
KICH	110.07	2.42	11.35	9.96	21.21	28.81
KIRC	233983.25	4.86	270.31	273.37	1445.95	1634.99
KIRP	6333196.216	21.73	2399.27	2373.97	8548.90	15488.37
LGG	8424102.257	22.09	1569.05	1288.19	3531.20	10269.79
LIHC	1478483.54	10.73	889.00	864.86	2312.95	5628.89
LUAD	20618742.8	26.69	4252.91	4756.55	8819.61	30047.28
LUSC	7845996.176	22.16	2415.54	2558.71	6506.50	17631.06
MESO	56329.84	4.37	161.71	173.62	1194.69	1438.82
OV	14579200.48	24.19	4860.35	5348.26	13364.62	33690.44
PAAD	87607.16	2.95	250.49	218.37	1093.72	1473.88
PCPG	106471.89	3.46	106.65	111.74	768.08	1001.99
PRAD	12528692.6	25.72	2111.75	2301.42	4903.29	16511.41
READ	3568809.075	16.99	1998.72	1554.24	11747.62	14794.83
SARC	965960.28	8.28	724.43	699.18	2546.29	2153.18
SKCM	2699142.101	18.02	1741.30	1931.21	10192.75	12996.01
STAD	23169148.34	26.77	3985.27	4535.03	7899.07	26889.59
TGCT	25679.1	1.63	45.57	44.94	263.08	372.76
THCA	15294868.83	25.41	3092.61	3355.18	6290.82	20889.21
THYM	323881.19	8.91	658.68	673.87	3243.35	4646.12
UCEC	24363489.34	28.04	2947.77	3840.96	9233.21	25321.13
UCS	10969.19	3.31	72.80	71.57	477.34	543.57
UVM	7948.31	1.87	17.74	38.16	259.66	295.59

Average		15.25	1766.52	1873.08	5078.75	12143.96
Difference			1751.26	106.56	3205.67	7065.21

Table S6: Run times for each cancer type and opt/parallel implementation for 4-hit comb

	GPU
**Cancer Type**	**Upper Triangular Mapping**
ACC	38369.30
BLCA	>15 days
BRCA	>15 days
CESC	>15 days
CHOL	80277.86
COAD	>15 days
DLBC	175638.66
ESCA	204610.67
GBM	>15 days
HNSC	>15 days
KICH	101.69
KIRC	486401.01
KIRP	>15 days
LGG	>15 days
LIHC	>15 days
LUAD	>15 days
LUSC	>15 days
MESO	84519.86
OV	>15 days
PAAD	211639.01
PCPG	291697.75
PRAD	>15 days
READ	>15 days
SARC	>15 days
SKCM	963632.13
STAD	>15 days
TGCT	193774.70
THCA	>15 days
THYM	270664.92
UCEC	>15 days
UCS	22075.00
UVM	34100.45

Table S7: 2-hit combinations identified for each cancer type.

Cancer	Gene 1	Gene 2	Number of Samples Covered	Fraction of Samples Covered
ACC	ENSG00000145113	ENSG00000173213	33	0.60
ACC	ENSG00000279804	ENSG00000227152	23	0.42
ACC	ENSG00000149531	ENSG00000100197	15	0.27
ACC	ENSG00000149531	ENSG00000173546	19	0.35
ACC	ENSG00000196126	ENSG00000239511	3	0.05
BLCA	ENSG00000184956	ENSG00000173213	138	0.51
BLCA	ENSG00000169862	ENSG00000149531	127	0.47
BLCA	ENSG00000172765	ENSG00000139687	37	0.14
BLCA	ENSG00000140836	ENSG00000124762	26	0.10
BLCA	ENSG00000005844	ENSG00000141232	16	0.06
BLCA	ENSG00000137776	ENSG00000068078	21	0.08
BLCA	ENSG00000156650	ENSG00000074356	12	0.04
BLCA	ENSG00000078237	ENSG00000196498	8	0.03
BLCA	ENSG00000137776	ENSG00000111879	9	0.03
BLCA	ENSG00000211650	ENSG00000170549	12	0.04
BLCA	ENSG00000165966	ENSG00000172164	4	0.01
BLCA	ENSG00000129315	ENSG00000099338	9	0.03
BLCA	ENSG00000005339	ENSG00000124253	8	0.03
BLCA	ENSG00000188039	ENSG00000147251	9	0.03
BLCA	ENSG00000219481	ENSG00000196275	5	0.02
BRCA	ENSG00000205277	ENSG00000184956	587	0.86
BRCA	ENSG00000149531	ENSG00000090512	93	0.14
BRCA	ENSG00000219481	ENSG00000227152	133	0.19
BRCA	ENSG00000149531	ENSG00000204172	186	0.27
BRCA	ENSG00000219481	ENSG00000173213	229	0.33
BRCA	ENSG00000141510	ENSG00000172785	42	0.06
BRCA	ENSG00000213512	ENSG00000139324	24	0.04
BRCA	ENSG00000171735	ENSG00000089091	7	0.01
CESC	ENSG00000184956	ENSG00000173213	149	0.68
CESC	ENSG00000205277	ENSG00000149531	131	0.60
CESC	ENSG00000205277	ENSG00000278662	107	0.49
CESC	ENSG00000174501	ENSG00000204583	71	0.33
CESC	ENSG00000197915	ENSG00000104140	7	0.03
CESC	ENSG00000237702	ENSG00000175170	19	0.09
CESC	ENSG00000143603	ENSG00000165118	3	0.01
CESC	ENSG00000126856	ENSG00000101298	4	0.02
CHOL	ENSG00000205277	ENSG00000149531	26	0.81

CHOL	ENSG00000127603	ENSG00000159433	8	0.25
CHOL	ENSG00000198128	ENSG00000239511	6	0.19
COAD	ENSG00000134982	ENSG00000149531	207	0.72
COAD	ENSG00000184956	ENSG00000173213	156	0.54
COAD	ENSG00000196074	ENSG00000112159	90	0.31
COAD	ENSG00000120314	ENSG00000143882	29	0.10
COAD	ENSG00000204130	ENSG00000169592	43	0.15
COAD	ENSG00000176542	ENSG00000133703	64	0.22
COAD	ENSG00000176542	ENSG00000133030	26	0.09
COAD	ENSG00000206503	ENSG00000089351	8	0.03
COAD	ENSG00000099290	ENSG00000109061	13	0.04
COAD	ENSG00000196581	ENSG00000166428	4	0.01
DLBC	ENSG00000149531	ENSG00000211899	23	0.62
DLBC	ENSG00000145113	ENSG00000173213	22	0.59
DLBC	ENSG00000170075	ENSG00000115844	11	0.30
ESCA	ENSG00000205277	ENSG00000141510	73	0.95
ESCA	ENSG00000266338	ENSG00000204583	16	0.21
HNSC	ENSG00000184956	ENSG00000173213	236	0.65
HNSC	ENSG00000169862	ENSG00000149531	158	0.43
HNSC	ENSG00000214324	ENSG00000184956	80	0.22
HNSC	ENSG00000141510	ENSG00000162927	52	0.14
HNSC	ENSG00000141510	ENSG00000166444	43	0.12
HNSC	ENSG00000253188	ENSG00000141510	49	0.13
HNSC	ENSG00000118160	ENSG00000183837	23	0.06
HNSC	ENSG00000148600	ENSG00000100321	9	0.02
HNSC	ENSG00000243811	ENSG00000131504	10	0.03
HNSC	ENSG00000151458	ENSG00000171936	5	0.01
HNSC	ENSG00000148219	ENSG00000260903	6	0.02
HNSC	ENSG00000133026	ENSG00000204880	4	0.01
HNSC	ENSG00000197429	ENSG00000106086	2	0.01
HNSC	ENSG00000197838	ENSG00000029639	3	0.01
KICH	ENSG00000138767	ENSG00000149531	5	0.83
KICH	ENSG00000145113	ENSG00000177692	2	0.33
KIRC	ENSG00000184956	ENSG00000173213	34	0.47
KIRC	ENSG00000149531	ENSG00000119707	28	0.38
KIRC	ENSG00000187545	ENSG00000134086	15	0.21
KIRC	ENSG00000149531	ENSG00000198754	16	0.22
KIRC	ENSG00000268043	ENSG00000063169	7	0.10
KIRC	ENSG00000270550	ENSG00000172236	5	0.07
KIRC	ENSG00000273136	ENSG00000091947	4	0.05

KIRP	ENSG00000184956	ENSG00000173213	97	0.56
KIRP	ENSG00000196436	ENSG00000149531	81	0.47
KIRP	ENSG00000152784	ENSG00000197272	26	0.15
KIRP	ENSG00000227152	ENSG00000145113	49	0.28
KIRP	ENSG00000138756	ENSG00000130675	14	0.08
KIRP	ENSG00000187840	ENSG00000149531	46	0.27
KIRP	ENSG00000070413	ENSG00000140795	4	0.02
KIRP	ENSG00000221923	ENSG00000115977	7	0.04
KIRP	ENSG00000136699	ENSG00000099331	6	0.03
KIRP	ENSG00000132780	ENSG00000139263	4	0.02
LIHC	ENSG00000149531	ENSG00000134775	146	0.63
LIHC	ENSG00000227152	ENSG00000184956	106	0.46
LIHC	ENSG00000184956	ENSG00000173213	105	0.45
LIHC	ENSG00000162843	ENSG00000175170	97	0.42
LIHC	ENSG00000113211	ENSG00000149531	68	0.29
LIHC	ENSG00000093000	ENSG00000275395	46	0.20
LIHC	ENSG00000177212	ENSG00000170370	24	0.10
LIHC	ENSG00000177212	ENSG00000182687	8	0.03
LIHC	ENSG00000160072	ENSG00000166206	2	0.01
LUAD	ENSG00000184956	ENSG00000173213	187	0.62
LUAD	ENSG00000169862	ENSG00000149531	158	0.52
LUAD	ENSG00000151846	ENSG00000133703	99	0.33
LUAD	ENSG00000141510	ENSG00000170396	50	0.17
LUAD	ENSG00000135077	ENSG00000141510	22	0.07
LUAD	ENSG00000204577	ENSG00000174514	15	0.05
LUAD	ENSG00000166049	ENSG00000198900	16	0.05
LUAD	ENSG00000205744	ENSG00000196924	8	0.03
LUAD	ENSG00000219481	ENSG00000007080	5	0.02
LUAD	ENSG00000104805	ENSG00000176406	7	0.02
LUAD	ENSG00000187634	ENSG00000152784	4	0.01
LUSC	ENSG00000184956	ENSG00000173213	131	0.60
LUSC	ENSG00000141510	ENSG00000149531	102	0.46
LUSC	ENSG00000141510	ENSG00000136531	39	0.18
LUSC	ENSG00000196126	ENSG00000171862	26	0.12
LUSC	ENSG00000143575	ENSG00000198597	15	0.07
LUSC	ENSG00000141510	ENSG00000121297	45	0.20
LUSC	ENSG00000170382	ENSG00000127863	4	0.02
LUSC	ENSG00000141510	ENSG00000092148	18	0.08
LUSC	ENSG00000130726	ENSG00000162896	4	0.02
LUSC	ENSG00000198216	ENSG00000103375	5	0.02
LUSC	ENSG00000100811	ENSG00000113396	3	0.01
LUSC	ENSG00000042781	ENSG00000182315	5	0.02
MESO	ENSG00000118762	ENSG00000174450	20	0.40

MESO	ENSG00000205277	ENSG00000185950	19	0.38
MESO	ENSG00000152192	ENSG00000161610	18	0.36
MESO	ENSG00000174450	ENSG00000165244	8	0.16
MESO	ENSG00000196581	ENSG00000198727	7	0.14
MESO	ENSG00000163959	ENSG00000100154	2	0.04
PAAD	ENSG00000169862	ENSG00000149531	86	0.87
PAAD	ENSG00000197915	ENSG00000173213	79	0.80
PAAD	ENSG00000121851	ENSG00000130561	2	0.02
PRAD	ENSG00000184956	ENSG00000173213	189	0.62
PRAD	ENSG00000169862	ENSG00000149531	140	0.46
PRAD	ENSG00000213516	ENSG00000121067	24	0.08
PRAD	ENSG00000242950	ENSG00000149531	86	0.28
PRAD	ENSG00000198502	ENSG00000227152	23	0.08
PRAD	ENSG00000242950	ENSG00000164976	8	0.03
PRAD	ENSG00000204442	ENSG00000187955	7	0.02
PRAD	ENSG00000074621	ENSG00000144619	12	0.04
PRAD	ENSG00000132964	ENSG00000141232	5	0.02
PRAD	ENSG00000103449	ENSG00000165392	12	0.04
PRAD	ENSG00000007923	ENSG00000167881	9	0.03
PRAD	ENSG00000198502	ENSG00000180878	3	0.01
PRAD	ENSG00000117228	ENSG00000103544	4	0.01
PRAD	ENSG00000159625	ENSG00000188152	9	0.03
PRAD	ENSG00000180357	ENSG00000187257	5	0.02
PRAD	ENSG00000260097	ENSG00000068308	3	0.01
PRAD	ENSG00000109758	ENSG00000161040	3	0.01
PRAD	ENSG00000155846	ENSG00000128011	10	0.03
READ	ENSG00000134982	ENSG00000149531	81	0.74
READ	ENSG00000197915	ENSG00000173213	59	0.54
READ	ENSG00000141510	ENSG00000101126	23	0.21
READ	ENSG00000168484	ENSG00000198786	9	0.08
READ	ENSG00000176542	ENSG00000130299	4	0.04
READ	ENSG00000157423	ENSG00000180176	3	0.03
READ	ENSG00000114270	ENSG00000131242	2	0.02
SARC	ENSG00000205277	ENSG00000149531	124	0.72
SARC	ENSG00000184956	ENSG00000173213	100	0.58
SARC	ENSG00000279457	ENSG00000205277	102	0.59
SARC	ENSG00000174501	ENSG00000204583	84	0.49
SARC	ENSG00000205277	ENSG00000278662	104	0.60
SARC	ENSG00000155657	ENSG00000181001	6	0.03
STAD	ENSG00000184956	ENSG00000173213	169	0.58
STAD	ENSG00000169862	ENSG00000149531	119	0.41
STAD	ENSG00000116251	ENSG00000178209	33	0.11

STAD	ENSG00000253188	ENSG00000141510	35	0.12
STAD	ENSG00000121297	ENSG00000141510	35	0.12
STAD	ENSG00000203786	ENSG00000171936	29	0.10
STAD	ENSG00000244482	ENSG00000243480	13	0.04
STAD	ENSG00000196240	ENSG00000140478	10	0.03
STAD	ENSG00000204442	ENSG00000173908	6	0.02
STAD	ENSG00000196126	ENSG00000204449	18	0.06
STAD	ENSG00000211896	ENSG00000074356	19	0.07
STAD	ENSG00000204479	ENSG00000104723	9	0.03
STAD	ENSG00000159387	ENSG00000129951	4	0.01
STAD	ENSG00000186815	ENSG00000248333	5	0.02
STAD	ENSG00000198837	ENSG00000156374	4	0.01
STAD	ENSG00000133216	ENSG00000178235	8	0.03
STAD	ENSG00000143631	ENSG00000197245	2	0.01
TGCT	ENSG00000104131	ENSG00000130528	67	0.61
TGCT	ENSG00000007923	ENSG00000149531	54	0.50
TGCT	ENSG00000134775	ENSG00000173213	38	0.35
TGCT	ENSG00000140836	ENSG00000274211	39	0.36
TGCT	ENSG00000197915	ENSG00000227152	37	0.34
THCA	ENSG00000184956	ENSG00000173213	192	0.60
THCA	ENSG00000169862	ENSG00000149531	138	0.43
THCA	ENSG00000186395	ENSG00000157764	64	0.20
THCA	ENSG00000269067	ENSG00000157764	44	0.14
THCA	ENSG00000197915	ENSG00000214324	56	0.18
THCA	ENSG00000130244	ENSG00000149531	51	0.16
THCA	ENSG00000112559	ENSG00000157764	34	0.11
THCA	ENSG00000142512	ENSG00000109046	10	0.03
THCA	ENSG00000149970	ENSG00000188921	4	0.01
THCA	ENSG00000122386	ENSG00000153207	6	0.02
THCA	ENSG00000198908	ENSG00000133773	11	0.03
THCA	ENSG00000268043	ENSG00000137161	5	0.02
THCA	ENSG00000116273	ENSG00000132024	3	0.01
THCA	ENSG00000159450	ENSG00000128829	9	0.03
THCA	ENSG00000161714	ENSG00000102401	7	0.02
THCA	ENSG00000269067	ENSG00000180592	5	0.02
THYM	ENSG00000205277	ENSG00000149531	63	0.80
THYM	ENSG00000279457	ENSG00000135976	55	0.70
THYM	ENSG00000174501	ENSG00000159140	7	0.09
UCEC	ENSG00000205277	ENSG00000149531	244	0.67
UCEC	ENSG00000184956	ENSG00000173213	204	0.56
UCEC	ENSG00000196126	ENSG00000171862	169	0.46
UCEC	ENSG00000205277	ENSG00000278662	143	0.39
UCEC	ENSG00000149531	ENSG00000183856	99	0.27

UCEC	ENSG00000143631	ENSG00000280363	14	0.04
UCEC	ENSG00000141510	ENSG00000196944	31	0.08
UCEC	ENSG00000116721	ENSG00000132840	12	0.03
UCEC	ENSG00000066056	ENSG00000244045	7	0.02
UCS	ENSG00000184956	ENSG00000173213	20	0.57
UCS	ENSG00000109670	ENSG00000141510	16	0.46
UCS	ENSG00000141510	ENSG00000149531	14	0.40
UCS	ENSG00000198837	ENSG00000101162	3	0.09
UCS	ENSG00000219481	ENSG00000110958	2	0.06
UVM	ENSG00000176148	ENSG00000101049	31	0.63
UVM	ENSG00000245680	ENSG00000088256	16	0.33
UVM	ENSG00000172765	ENSG00000173213	11	0.22
UVM	ENSG00000100365	ENSG00000221866	5	0.10
UVM	ENSG00000149531	ENSG00000169862	20	0.41
UVM	ENSG00000172869	ENSG00000104131	1	0.02

Table S8: 3-hit combinations identified for each cancer type.

Cancer	Gene 1	Gene 2	Gene 3	Number of Samples Covered	Fraction of Samples covered
ACC	ENSG00000145113	ENSG00000184956	ENSG00000173213	33	0.60
ACC	ENSG00000279804	ENSG00000127418	ENSG00000170442	27	0.49
ACC	ENSG00000198128	ENSG00000276119	ENSG00000149531	12	0.22
ACC	ENSG00000130711	ENSG00000130558	ENSG00000093000	10	0.18
BLCA	ENSG00000169862	ENSG00000184956	ENSG00000149531	127	0.47
BLCA	ENSG00000197915	ENSG00000184956	ENSG00000173213	119	0.44
BLCA	ENSG00000141510	ENSG00000104941	ENSG00000211896	43	0.16
BLCA	ENSG00000104974	ENSG00000116350	ENSG00000121879	33	0.12
BLCA	ENSG00000169047	ENSG00000172765	ENSG00000147050	28	0.10
BLCA	ENSG00000151846	ENSG00000133958	ENSG00000068078	18	0.07
BLCA	ENSG00000129315	ENSG00000141510	ENSG00000131042	24	0.09
BLCA	ENSG00000219481	ENSG00000126705	ENSG00000141232	8	0.03
BLCA	ENSG00000065526	ENSG00000141510	ENSG00000196943	17	0.06
BLCA	ENSG00000158290	ENSG00000198216	ENSG00000243156	9	0.03
BLCA	ENSG00000163959	ENSG00000188155	ENSG00000144136	11	0.04
BLCA	ENSG00000211896	ENSG00000103449	ENSG00000240403	24	0.09
BRCA	ENSG00000145113	ENSG00000205277	ENSG00000184956	549	0.80
BRCA	ENSG00000184956	ENSG00000149531	ENSG00000170471	121	0.18
BRCA	ENSG00000184956	ENSG00000226761	ENSG00000173213	259	0.38
BRCA	ENSG00000151846	ENSG00000149531	ENSG00000211725	221	0.32
BRCA	ENSG00000226761	ENSG00000215454	ENSG00000090512	70	0.10
BRCA	ENSG00000226761	ENSG00000196436	ENSG00000249931	21	0.03
BRCA	ENSG00000184956	ENSG00000149531	ENSG00000137210	68	0.10
BRCA	ENSG00000184956	ENSG00000174450	ENSG00000172785	26	0.04
CESC	ENSG00000145113	ENSG00000184956	ENSG00000173213	138	0.63
CESC	ENSG00000205277	ENSG00000184956	ENSG00000149531	131	0.60
CESC	ENSG00000205277	ENSG00000184956	ENSG00000278662	107	0.49
CESC	ENSG00000155657	ENSG00000184956	ENSG00000173213	118	0.54
CESC	ENSG00000197915	ENSG00000174501	ENSG00000204583	62	0.28
CESC	ENSG00000198502	ENSG00000237541	ENSG00000104140	6	0.03
CESC	ENSG00000174501	ENSG00000010438	ENSG00000237702	25	0.11
CESC	ENSG00000198502	ENSG00000046889	ENSG00000173166	4	0.02
CESC	ENSG00000154556	ENSG00000137185	ENSG00000167548	1	0.00
CHOL	ENSG00000205277	ENSG00000184956	ENSG00000149531	26	0.81
CHOL	ENSG00000174501	ENSG00000104974	ENSG00000115844	11	0.34
CHOL	ENSG00000069712	ENSG00000273136	ENSG00000164037	2	0.06

COAD	ENSG00000184956	ENSG00000134982	ENSG00000149531	206	0.71
COAD	ENSG00000184956	ENSG00000173213	ENSG00000055609	149	0.52
COAD	ENSG00000234745	ENSG00000275395	ENSG00000138674	102	0.35
COAD	ENSG00000234745	ENSG00000204130	ENSG00000143882	31	0.11
COAD	ENSG00000268043	ENSG00000156113	ENSG00000100888	30	0.10
COAD	ENSG00000162620	ENSG00000163872	ENSG00000167306	29	0.10
COAD	ENSG00000083642	ENSG00000121879	ENSG00000181585	29	0.10
COAD	ENSG00000083642	ENSG00000184634	ENSG00000177427	9	0.03
COAD	ENSG00000184956	ENSG00000157423	ENSG00000172725	8	0.03
COAD	ENSG00000232629	ENSG00000204007	ENSG00000204130	5	0.02
DLBC	ENSG00000136939	ENSG00000149531	ENSG00000134775	25	0.68
DLBC	ENSG00000145113	ENSG00000185567	ENSG00000211899	23	0.62
DLBC	ENSG00000116690	ENSG00000198700	ENSG00000182218	3	0.08
ESCA	ENSG00000205277	ENSG00000184956	ENSG00000141510	73	0.95
ESCA	ENSG00000266338	ENSG00000145113	ENSG00000204583	16	0.21
HNSC	ENSG00000197915	ENSG00000184956	ENSG00000173213	222	0.61
HNSC	ENSG00000169862	ENSG00000184956	ENSG00000149531	158	0.43
HNSC	ENSG00000141510	ENSG00000104941	ENSG00000211896	98	0.27
HNSC	ENSG00000214324	ENSG00000184956	ENSG00000151846	71	0.20
HNSC	ENSG00000141510	ENSG00000104974	ENSG00000090402	47	0.13
HNSC	ENSG00000141510	ENSG00000104974	ENSG00000179869	74	0.20
HNSC	ENSG00000139372	ENSG00000140836	ENSG00000165899	19	0.05
HNSC	ENSG00000185567	ENSG00000159023	ENSG00000113212	14	0.04
HNSC	ENSG00000198837	ENSG00000138756	ENSG00000085514	12	0.03
HNSC	ENSG00000158865	ENSG00000004455	ENSG00000211896	30	0.08
HNSC	ENSG00000197915	ENSG00000221859	ENSG00000146909	9	0.02
HNSC	ENSG00000154358	ENSG00000169894	ENSG00000189037	2	0.01
KICH	ENSG00000143226	ENSG00000145113	ENSG00000149531	6	1.00
KIRC	ENSG00000010438	ENSG00000184956	ENSG00000173213	33	0.45
KIRC	ENSG00000055609	ENSG00000149531	ENSG00000119707	27	0.37
KIRC	ENSG00000219481	ENSG00000197915	ENSG00000134086	16	0.22
KIRC	ENSG00000219481	ENSG00000149531	ENSG00000204501	14	0.19
KIRC	ENSG00000137414	ENSG00000120800	ENSG00000106536	7	0.10
KIRC	ENSG00000143631	ENSG00000143226	ENSG00000160298	5	0.07
KIRP	ENSG00000010438	ENSG00000184956	ENSG00000173213	89	0.52
KIRP	ENSG00000184956	ENSG00000196436	ENSG00000149531	81	0.47
KIRP	ENSG00000153165	ENSG00000140836	ENSG00000175170	41	0.24
KIRP	ENSG00000136939	ENSG00000149531	ENSG00000198502	63	0.37
KIRP	ENSG00000145113	ENSG00000237541	ENSG00000183337	30	0.17
KIRP	ENSG00000239961	ENSG00000110400	ENSG00000125817	8	0.05
KIRP	ENSG00000187676	ENSG00000211970	ENSG00000101004	5	0.03

KIRP	ENSG00000256453	ENSG00000170835	ENSG00000139985	7	0.04
KIRP	ENSG00000154358	ENSG00000261150	ENSG00000237702	3	0.02
LIHC	ENSG00000184956	ENSG00000149531	ENSG00000134775	146	0.63
LIHC	ENSG00000227152	ENSG00000145113	ENSG00000184956	102	0.44
LIHC	ENSG00000184956	ENSG00000196126	ENSG00000173213	87	0.38
LIHC	ENSG00000145113	ENSG00000157654	ENSG00000184634	58	0.25
LIHC	ENSG00000113211	ENSG00000184956	ENSG00000149531	68	0.29
LIHC	ENSG00000177212	ENSG00000141510	ENSG00000165055	41	0.18
LIHC	ENSG00000172765	ENSG00000157212	ENSG00000148204	31	0.13
LIHC	ENSG00000177212	ENSG00000222038	ENSG00000183747	32	0.14
LUAD	ENSG00000197915	ENSG00000184956	ENSG00000173213	172	0.57
LUAD	ENSG00000169862	ENSG00000184956	ENSG00000149531	158	0.52
LUAD	ENSG00000184956	ENSG00000151846	ENSG00000133703	99	0.33
LUAD	ENSG00000116147	ENSG00000155657	ENSG00000141510	43	0.14
LUAD	ENSG00000211896	ENSG00000079385	ENSG00000182704	38	0.13
LUAD	ENSG00000010438	ENSG00000140968	ENSG00000203786	19	0.06
LUAD	ENSG00000171680	ENSG00000140836	ENSG00000149084	13	0.04
LUAD	ENSG00000273136	ENSG00000121297	ENSG00000227234	20	0.07
LUAD	ENSG00000273136	ENSG00000197915	ENSG00000169551	6	0.02
LUAD	ENSG00000121753	ENSG00000117069	ENSG00000143630	5	0.02
LUSC	ENSG00000184956	ENSG00000173213	ENSG00000197915	123	0.56
LUSC	ENSG00000184956	ENSG00000141510	ENSG00000149531	102	0.46
LUSC	ENSG00000154222	ENSG00000256188	ENSG00000141510	48	0.22
LUSC	ENSG00000187537	ENSG00000141510	ENSG00000150275	29	0.13
LUSC	ENSG00000155657	ENSG00000177432	ENSG00000198597	17	0.08
LUSC	ENSG00000155657	ENSG00000146112	ENSG00000170959	10	0.05
LUSC	ENSG00000141510	ENSG00000104941	ENSG00000095917	27	0.12
LUSC	ENSG00000155657	ENSG00000005379	ENSG00000171365	8	0.04
LUSC	ENSG00000198216	ENSG00000168286	ENSG00000100889	5	0.02
LUSC	ENSG00000114861	ENSG00000187527	ENSG00000196126	8	0.04
MESO	ENSG00000118762	ENSG00000205277	ENSG00000151846	21	0.42
MESO	ENSG00000145113	ENSG00000226761	ENSG00000152192	21	0.42
MESO	ENSG00000196581	ENSG00000204983	ENSG00000179532	17	0.34
MESO	ENSG00000163959	ENSG00000070756	ENSG00000225614	6	0.12
MESO	ENSG00000232629	ENSG00000106536	ENSG00000180592	3	0.06
PAAD	ENSG00000169862	ENSG00000055609	ENSG00000149531	86	0.87
PAAD	ENSG00000143603	ENSG00000143882	ENSG00000145216	60	0.61
PRAD	ENSG00000184956	ENSG00000197915	ENSG00000173213	174	0.57
PRAD	ENSG00000169862	ENSG00000184956	ENSG00000149531	140	0.46
PRAD	ENSG00000196187	ENSG00000211896	ENSG00000125498	43	0.14
PRAD	ENSG00000177432	ENSG00000184956	ENSG00000149531	69	0.23

PRAD	ENSG00000213516	ENSG00000148773	ENSG00000121067	21	0.07
PRAD	ENSG00000148773	ENSG00000074356	ENSG00000198885	17	0.06
PRAD	ENSG00000121057	ENSG00000125498	ENSG00000077264	12	0.04
PRAD	ENSG00000256436	ENSG00000198908	ENSG00000079805	11	0.04
PRAD	ENSG00000187545	ENSG00000099995	ENSG00000125657	18	0.06
PRAD	ENSG00000106823	ENSG00000211972	ENSG00000204479	11	0.04
PRAD	ENSG00000148773	ENSG00000099917	ENSG00000205856	9	0.03
PRAD	ENSG00000151846	ENSG00000135341	ENSG00000160957	5	0.02
PRAD	ENSG00000179912	ENSG00000156030	ENSG00000124493	4	0.01
PRAD	ENSG00000107736	ENSG00000204442	ENSG00000158966	2	0.01
PRAD	ENSG00000148773	ENSG00000196539	ENSG00000155034	3	0.01
READ	ENSG00000134982	ENSG00000184956	ENSG00000149531	81	0.74
READ	ENSG00000197915	ENSG00000055609	ENSG00000173213	59	0.54
READ	ENSG00000204130	ENSG00000168484	ENSG00000083642	12	0.11
READ	ENSG00000184956	ENSG00000141510	ENSG00000101126	23	0.21
READ	ENSG00000204130	ENSG00000176058	ENSG00000140839	6	0.06
READ	ENSG00000160299	ENSG00000177103	ENSG00000213967	3	0.03
SARC	ENSG00000205277	ENSG00000184956	ENSG00000149531	124	0.72
SARC	ENSG00000145113	ENSG00000184956	ENSG00000173213	96	0.56
SARC	ENSG00000279457	ENSG00000145113	ENSG00000205277	98	0.57
SARC	ENSG00000174501	ENSG00000184956	ENSG00000204583	84	0.49
SARC	ENSG00000155657	ENSG00000205277	ENSG00000278662	67	0.39
SARC	ENSG00000155657	ENSG00000169047	ENSG00000175170	15	0.09
STAD	ENSG00000197915	ENSG00000184956	ENSG00000173213	152	0.52
STAD	ENSG00000169862	ENSG00000184956	ENSG00000149531	119	0.41
STAD	ENSG00000143631	ENSG00000211896	ENSG00000141510	63	0.22
STAD	ENSG00000116251	ENSG00000155657	ENSG00000151846	38	0.13
STAD	ENSG00000168702	ENSG00000211947	ENSG00000141510	45	0.15
STAD	ENSG00000018625	ENSG00000130779	ENSG00000096384	32	0.11
STAD	ENSG00000198128	ENSG00000155657	ENSG00000173213	65	0.22
STAD	ENSG00000141510	ENSG00000109919	ENSG00000182985	34	0.12
STAD	ENSG00000127603	ENSG00000118160	ENSG00000144893	20	0.07
STAD	ENSG00000188906	ENSG00000187688	ENSG00000137492	7	0.02
STAD	ENSG00000163040	ENSG00000166164	ENSG00000167608	11	0.04
STAD	ENSG00000143631	ENSG00000099381	ENSG00000140795	18	0.06
STAD	ENSG00000204577	ENSG00000173153	ENSG00000162779	6	0.02
STAD	ENSG00000143631	ENSG00000143575	ENSG00000197245	1	0.00
TGCT	ENSG00000184956	ENSG00000104131	ENSG00000130528	67	0.61
TGCT	ENSG00000007923	ENSG00000145113	ENSG00000243156	57	0.52
TGCT	ENSG00000204983	ENSG00000156017	ENSG00000065970	43	0.39
TGCT	ENSG00000005073	ENSG00000157212	ENSG00000139613	17	0.16
THCA	ENSG00000055609	ENSG00000184956	ENSG00000173213	172	0.54

THCA	ENSG00000169862	ENSG00000184956	ENSG00000149531	138	0.43
THCA	ENSG00000070756	ENSG00000043355	ENSG00000157764	74	0.23
THCA	ENSG00000157764	ENSG00000171680	ENSG00000074621	64	0.20
THCA	ENSG00000116539	ENSG00000211896	ENSG00000131042	32	0.10
THCA	ENSG00000256436	ENSG00000211895	ENSG00000105697	14	0.04
THCA	ENSG00000149531	ENSG00000005022	ENSG00000164309	53	0.17
THCA	ENSG00000151846	ENSG00000157764	ENSG00000211731	52	0.16
THCA	ENSG00000186409	ENSG00000255408	ENSG00000211896	27	0.08
THCA	ENSG00000050438	ENSG00000239961	ENSG00000151690	7	0.02
THCA	ENSG00000197915	ENSG00000164938	ENSG00000184923	6	0.02
THCA	ENSG00000133226	ENSG00000197915	ENSG00000105388	4	0.01
THCA	ENSG00000115464	ENSG00000172985	ENSG00000164037	2	0.01
THYM	ENSG00000205277	ENSG00000184956	ENSG00000149531	63	0.80
THYM	ENSG00000279457	ENSG00000219481	ENSG00000135976	46	0.58
THYM	ENSG00000109536	ENSG00000263001	ENSG00000205277	15	0.19
UCEC	ENSG00000205277	ENSG00000184956	ENSG00000149531	244	0.67
UCEC	ENSG00000184956	ENSG00000173213	ENSG00000055609	177	0.48
UCEC	ENSG00000171862	ENSG00000184956	ENSG00000181143	176	0.48
UCEC	ENSG00000205277	ENSG00000141510	ENSG00000010438	112	0.31
UCEC	ENSG00000256436	ENSG00000149531	ENSG00000183856	88	0.24
UCEC	ENSG00000197915	ENSG00000205277	ENSG00000211896	136	0.37
UCEC	ENSG00000205277	ENSG00000153165	ENSG00000198947	97	0.27
UCEC	ENSG00000131018	ENSG00000253534	ENSG00000150990	23	0.06
UCEC	ENSG00000261456	ENSG00000180592	ENSG00000215182	21	0.06
UCS	ENSG00000196126	ENSG00000184956	ENSG00000173213	19	0.54
UCS	ENSG00000196126	ENSG00000141510	ENSG00000187823	15	0.43
UCS	ENSG00000109670	ENSG00000169862	ENSG00000141510	12	0.34
UCS	ENSG00000198502	ENSG00000136939	ENSG00000133703	2	0.06
UVM	ENSG00000184956	ENSG00000176148	ENSG00000101049	31	0.63
UVM	ENSG00000211947	ENSG00000204983	ENSG00000163930	13	0.27
UVM	ENSG00000162927	ENSG00000104974	ENSG00000100271	18	0.37
UVM	ENSG00000156052	ENSG00000101040	ENSG00000115594	4	0.08
UVM	ENSG00000172765	ENSG00000237541	ENSG00000088256	4	0.08

Table S9: 4-hit combinations identified for each cancer type.

Cancer	Gene 1	Gene 2	Gene 3	Gene 4
ACC	ENSG00000145113	ENSG00000127418	ENSG00000184956	ENSG00000173213
ACC	ENSG00000279804	ENSG00000197915	ENSG00000104972	ENSG00000149531
ACC	ENSG00000204525	ENSG00000130558	ENSG00000184956	ENSG00000127507
ACC	ENSG00000279804	ENSG00000143226	ENSG00000143226	ENSG00000227152
CHOL	ENSG00000145113	ENSG00000205277	ENSG00000184956	ENSG00000149531
CHOL	ENSG00000184956	ENSG00000256436	ENSG00000181143	ENSG00000173213
CHOL	ENSG00000198128	ENSG00000215182	ENSG00000244482	ENSG00000186566
CHOL	ENSG00000007923	ENSG00000127481	ENSG00000142661	ENSG00000237702
DLBC	ENSG00000145113	ENSG00000136939	ENSG00000149531	ENSG00000134775
DLBC	ENSG00000143631	ENSG00000165055	ENSG00000070756	ENSG00000149531
DLBC	ENSG00000234745	ENSG00000104974	ENSG00000151327	ENSG00000151612
DLBC	ENSG00000163531	ENSG00000168016	ENSG00000172752	ENSG00000127418
ESCA	ENSG00000145113	ENSG00000205277	ENSG0000184956	ENSG00000141510
ESCA	ENSG00000205277	ENSG00000149531	ENSG00000104974	ENSG00000153165
ESCA	ENSG00000205038	ENSG00000204501	ENSG00000269713	ENSG00000148357
KICH	ENSG00000138767	ENSG00000131042	ENSG00000239961	ENSG00000149531
KICH	ENSG00000213516	ENSG00000143226	ENSG00000214595	ENSG00000106665
KIRC	ENSG00000010438	ENSG00000184956	ENSG00000104974	ENSG00000173213
KIRC	ENSG00000197915	ENSG00000055609	ENSG00000149531	ENSG00000158987
KIRC	ENSG00000143631	ENSG00000151946	ENSG00000239998	ENSG00000149531
KIRC	ENSG00000219481	ENSG00000134086	ENSG00000010438	ENSG00000131042
KIRC	ENSG00000204120	ENSG00000100604	ENSG00000243156	ENSG00000172943
KIRC	ENSG00000004455	ENSG00000155657	ENSG00000003989	ENSG00000173157
KIRC	ENSG00000213516	ENSG00000197915	ENSG00000139985	ENSG00000274349
MESO	ENSG00000049618	ENSG00000205277	ENSG00000184956	ENSG00000151846
MESO	ENSG00000145113	ENSG00000226761	ENSG00000151846	ENSG00000152192
MESO	ENSG00000184956	ENSG00000184185	ENSG00000055609	ENSG00000223638
MESO	ENSG00000196126	ENSG00000049618	ENSG00000100811	ENSG00000124813
MESO	ENSG00000079308	ENSG00000147234	ENSG00000060069	ENSG00000106511
MESO	ENSG00000010438	ENSG00000104974	ENSG00000100354	ENSG00000180592
PAAD	ENSG00000169862	ENSG00000148773	ENSG00000184956	ENSG00000173213
PAAD	ENSG00000169862	ENSG00000055609	ENSG00000137776	ENSG00000149531
PAAD	ENSG00000204479	ENSG00000219481	ENSG00000184677	ENSG00000130561
PCPG	ENSG00000055609	ENSG00000184956	ENSG00000173213	ENSG00000104972
PCPG	ENSG00000197915	ENSG00000169862	ENSG00000184956	ENSG00000149531
PCPG	ENSG00000196126	ENSG00000256188	ENSG00000149531	ENSG00000204442

PCPG	ENSG00000100346	ENSG00000253534	ENSG00000187840	ENSG00000172765
PCPG	ENSG00000196187	ENSG00000243772	ENSG00000165757	ENSG00000099995
PCPG	ENSG00000196126	ENSG00000226761	ENSG00000176231	ENSG00000143217
PCPG	ENSG00000226761	ENSG00000100207	ENSG00000165568	ENSG00000172765
PCPG	ENSG00000206503	ENSG00000242950	ENSG00000043355	ENSG00000146414
PCPG	ENSG00000184956	ENSG00000104941	ENSG00000172236	ENSG00000099957
PCPG	ENSG00000213516	ENSG00000197915	ENSG00000155846	ENSG00000211958
PCPG	ENSG00000165757	ENSG00000140839	ENSG00000115468	ENSG00000184634
PCPG	ENSG00000142599	ENSG00000273136	ENSG00000197915	ENSG00000124429
SKCM	ENSG00000197915	ENSG00000184956	ENSG00000104972	ENSG00000173213
SKCM	ENSG00000169862	ENSG00000196126	ENSG00000104974	ENSG00000149531
SKCM	ENSG00000157764	ENSG00000140836	ENSG00000104972	ENSG00000187840
SKCM	ENSG00000176771	ENSG00000138829	ENSG00000234745	ENSG00000131042
SKCM	ENSG00000118160	ENSG00000239998	ENSG00000187688	ENSG00000262655
SKCM	ENSG00000171680	ENSG00000147724	ENSG00000160602	ENSG00000214063
SKCM	ENSG00000187545	ENSG00000219481	ENSG00000049089	ENSG00000181867
TGCT	ENSG00000177212	ENSG00000145113	ENSG00000256436	ENSG00000104131
TGCT	ENSG00000184956	ENSG00000149531	ENSG00000099917	ENSG00000169184
TGCT	ENSG00000153165	ENSG00000055609	ENSG00000156017	ENSG00000166049
TGCT	ENSG00000157212	ENSG00000167110	ENSG00000184956	ENSG00000013573
TGCT	ENSG00000007923	ENSG00000126705	ENSG00000072694	ENSG00000237541
THYM	ENSG00000145113	ENSG00000205277	ENSG00000184956	ENSG00000149531
THYM	ENSG00000205277	ENSG00000184956	ENSG00000256436	ENSG00000250844
THYM	ENSG00000279457	ENSG00000219481	ENSG00000174501	ENSG00000153165
THYM	ENSG00000187545	ENSG00000186409	ENSG00000116774	ENSG00000119777
UCS	ENSG00000196126	ENSG00000184956	ENSG00000141510	ENSG00000173213
UCS	ENSG00000196126	ENSG00000184956	ENSG00000141510	ENSG00000187823
UCS	ENSG00000141510	ENSG00000104972	ENSG00000005022	ENSG00000211896
UCS	ENSG00000117983	ENSG00000184677	ENSG00000110274	ENSG00000135740
UCS	ENSG00000141510	ENSG00000099917	ENSG00000075886	ENSG00000154222
UVM	ENSG00000162927	ENSG00000184956	ENSG00000176148	ENSG00000100365
UVM	ENSG00000055609	ENSG00000133101	ENSG00000245680	ENSG00000100271
UVM	ENSG00000184956	ENSG00000256436	ENSG00000173213	ENSG00000169862
UVM	ENSG00000211727	ENSG00000125498	ENSG00000169047	ENSG00000088256
UVM	ENSG00000112079	ENSG00000010438	ENSG00000156052	ENSG00000196187
UVM	ENSG00000143882	ENSG00000206503	ENSG00000204983	ENSG00000130733

Table S10: Sensitivity and Specificity for each cancer type, showing calculation details for 2-hit combinations.

		Training Set								Test Set									
		Tumor Samples				Normal Samples				Tumor Samples					Normal Samples				
Cancer Type	#Combinations	True Positives	False Negatives	Total	Sensitivity	True Negatives	False Positives	Total	Specificity	True Positives	False Negatives	Total	Sensitivity	95% CI	True Negatives	False Positives	Total	Specificity	95% CI
ACC	5	55	0	55	100%	256	0	256	100%	23	2	25	92%	74-99%	75	2	77	97%	91-100%
BLCA	15	269	0	269	100%	256	0	256	100%	76	23	99	77%	67-85%	61	16	77	79%	68-88%
BRCA	8	684	0	684	100%	248	8	256	97%	220	7	227	97%	94-99%	70	7	77	91%	82-96%
CESC	8	218	0	218	100%	256	0	256	100%	48	8	56	86%	74-94%	76	1	77	99%	93-100%
CHOL	3	32	0	32	100%	256	0	256	100%	10	2	12	83%	52-98%	77	0	77	100%	95-100%
COAD	10	289	0	289	100%	256	0	256	100%	90	6	96	94%	87-98%	70	7	77	91%	82-96%
DLBC	3	37	0	37	100%	256	0	256	100%	5	1	6	83%	36-100%	77	0	77	100%	95-100%
ESCA	2	77	0	77	100%	255	1	256	100%	32	2	34	94%	80-99%	77	0	77	100%	95-100%
GBM	9	259	0	259	100%	256	0	256	100%	67	5	72	93%	85-98%	72	5	77	94%	85-98%
HNSC	14	364	0	364	100%	256	0	256	100%	98	8	106	92%	86-97%	67	10	77	87%	77-94%
KICH	2	6	0	6	100%	256	0	256	100%	3	0	3	100%	29-100%	76	1	77	99%	93-100%
KIRC	7	73	0	73	100%	256	0	256	100%	11	4	15	73%	45-92%	72	5	77	94%	85-98%
KIRP	10	172	0	172	100%	256	0	256	100%	54	2	56	96%	88-100%	70	7	77	91%	82-96%
LGG	11	363	0	363	100%	252	4	256	98%	112	4	116	97%	91-99%	69	8	77	90%	81-95%
LIHC	9	232	0	232	100%	255	1	256	100%	75	5	80	94%	86-98%	69	8	77	90%	81-95%
LUAD	11	301	0	301	100%	256	0	256	100%	92	16	108	85%	77-91%	65	12	77	84%	74-92%
LUSC	12	220	0	220	100%	256	0	256	100%	74	11	85	87%	78-93%	72	5	77	94%	85-98%
MESO	6	50	0	50	100%	256	0	256	100%	12	7	19	63%	38-84%	77	0	77	100%	95-100%
OV	8	226	0	226	100%	256	0	256	100%	84	7	91	92%	85-97%	76	1	77	99%	93-100%
PAAD	3	99	0	99	100%	256	0	256	100%	50	0	50	100%	93-100%	74	3	77	96%	89-99%
PRAD	18	306	0	306	100%	256	0	256	100%	95	20	115	83%	74-89%	70	7	77	91%	82-96%
READ	7	109	0	109	100%	256	0	256	100%	25	8	33	76%	58-89%	71	6	77	92%	84-97%
SARC	6	172	0	172	100%	256	0	256	100%	47	0	47	100%	92-100%	76	1	77	99%	93-100%
STAD	17	292	0	292	100%	256	0	256	100%	82	14	96	85%	77-92%	67	10	77	87%	77-94%
TGCT	5	109	0	109	100%	256	0	256	100%	37	3	40	92%	80-98%	74	3	77	96%	89-99%
THCA	16	320	0	320	100%	256	0	256	100%	91	10	101	90%	83-95%	70	7	77	91%	82-96%

THYM	3	79	0	79	100%	256	0	256	100%	24	4	28	86%	67-96%	76	1	77	99%	93-100%
UCEC	9	365	0	365	100%	256	0	256	100%	122	8	130	94%	88-97%	73	4	77	95%	87-99%
UCS	5	35	0	35	100%	256	0	256	100%	10	1	11	91%	59-100%	75	2	77	97%	91-100%
UVM	6	49	0	49	100%	256	0	256	100%	20	4	24	83%	63-95%	71	6	77	92%	84-97%
Total	248	5862	0	5862	100%	7666	14	7680	100%	1789	192	1981	90%	89-92%	2165	145	2310	94%	93-95%

Table S11: Sensitivity and Specificity for each cancer type, showing calculation details for 3-hit combinations

		Training Set								Test Set									
		Tumor Samples				Normal Samples				Tumor Samples					Normal Samples				
Cancer Type	#Combinations	True Positives	False Negatives	Total	Sensitivity	True Negatives	False Positives	Total	Specifcity	True Positives	False Negatives	Total	Sensitivity	95% CI	True Negatives	False Positives	Total	Specifcity	95% CI
ACC	4	55	0	55	100%	256	0	256	100%	22	3	25	88%	69-97%	76	1	77	99%	93-100%
BLCA	12	269	0	269	100%	256	0	256	100%	82	17	99	83%	74-90%	67	10	77	87%	77-94%
BRCA	8	684	0	684	100%	248	8	256	97%	216	11	227	95%	91-98%	70	7	77	91%	82-96%
CESC	9	218	0	218	100%	256	0	256	100%	48	8	56	86%	74-94%	77	0	77	100%	95-100%
CHOL	3	32	0	32	100%	256	0	256	100%	10	2	12	83%	52-98%	75	2	77	97%	91-100%
COAD	10	289	0	289	100%	256	0	256	100%	91	5	96	95%	88-98%	70	7	77	91%	82-96%
DLBC	3	37	0	37	100%	256	0	256	100%	3	3	6	50%	12-88%	77	0	77	100%	95-100%
ESCA	2	77	0	77	100%	255	1	256	100%	32	2	34	94%	80-99%	77	0	77	100%	95-100%
GBM	10	259	0	259	100%	256	0	256	100%	66	6	72	92%	83-97%	70	7	77	91%	82-96%
HNSC	12	364	0	364	100%	256	0	256	100%	97	9	106	92%	84-96%	71	6	77	92%	84-97%
KICH	1	6	0	6	100%	256	0	256	100%	0	3	3	0%	0-71%	75	2	77	97%	91-100%
KIRC	6	73	0	73	100%	256	0	256	100%	11	4	15	73%	45-92%	72	5	77	94%	85-98%
KIRP	9	172	0	172	100%	256	0	256	100%	49	7	56	88%	76-95%	68	9	77	88%	79-95%
LGG	11	363	0	363	100%	254	2	256	99%	107	9	116	92%	86-96%	69	8	77	90%	81-95%
LIHC	8	232	0	232	100%	255	1	256	100%	80	0	80	100%	95-100%	72	5	77	94%	85-98%
LUAD	10	301	0	301	100%	256	0	256	100%	95	13	108	88%	80-93%	66	11	77	86%	76-93%
LUSC	10	220	0	220	100%	256	0	256	100%	75	10	85	88%	79-94%	68	9	77	88%	79-95%
MESO	5	50	0	50	100%	256	0	256	100%	12	7	19	63%	38-84%	74	3	77	96%	89-99%
OV	8	226	0	226	100%	256	0	256	100%	80	11	91	88%	79-94%	76	1	77	99%	93-100%
PAAD	2	99	0	99	100%	256	0	256	100%	47	3	50	94%	83-99%	75	2	77	97%	91-100%
PRAD	15	306	0	306	100%	256	0	256	100%	96	19	115	83%	75-90%	60	17	77	78%	67-87%
READ	6	109	0	109	100%	256	0	256	100%	28	5	33	85%	68-95%	72	5	77	94%	85-98%
SARC	6	172	0	172	100%	256	0	256	100%	46	1	47	98%	89-100%	75	2	77	97%	91-100%
STAD	14	292	0	292	100%	256	0	256	100%	83	13	96	86%	78-93%	66	11	77	86%	76-93%
TGCT	4	109	0	109	100%	256	0	256	100%	33	7	40	82%	67-93%	72	5	77	94%	85-98%
THCA	13	320	0	320	100%	256	0	256	100%	88	13	101	87%	79-93%	69	8	77	90%	81-95%

THYM	3	79	0	79	100%	256	0	256	100%	24	4	28	86%	67-96%	76	1	77	99%	93-100%
UCEC	9	365	0	365	100%	256	0	256	100%	124	6	130	95%	90-98%	73	4	77	95%	87-99%
UCS	4	35	0	35	100%	256	0	256	100%	8	3	11	73%	39-94%	76	1	77	99%	93-100%
UVM	5	49	0	49	100%	256	0	256	100%	21	3	24	88%	68-97%	72	5	77	94%	85-98%
Total	222	5862	0	5862	100%	7668	12	7680	100%	1774	207	1981	90%	88-91%	2156	154	2310	93%	92-94%
